# Inverse localization of earliest cardiac activation sites from activation maps based on the viscous Eikonal equation

**DOI:** 10.1007/s00285-019-01419-3

**Published:** 2019-08-31

**Authors:** Karl Kunisch, Aurel Neic, Gernot Plank, Philip Trautmann

**Affiliations:** 1 Heinrichstraße 36, 8010 Graz, Austria; 2 Auenbruggerplatz 2, 8036 Graz, Austria

**Keywords:** Shape optimization, Nonlinear elliptic PDEs, Inverse problems, Electro physiology, 49Q10, 35J60, 49N45, 92C50

## Abstract

In this study we propose a novel method for identifying the locations of earliest activation in the human left ventricle from activation maps measured at the epicardial surface. Electrical activation is modeled based on the viscous Eikonal equation. The sites of earliest activation are identified by solving a minimization problem. Arbitrary initial locations are assumed, which are then modified based on a shape derivative based perturbation field until a minimal mismatch between the computed and the given activation maps on the epicardial surface is achieved. The proposed method is tested in two numerical benchmarks, a generic 2D unit-square benchmark, and an anatomically accurate MRI-derived 3D human left ventricle benchmark to demonstrate potential utility in a clinical context. For unperturbed input data, our localization method is able to accurately reconstruct the earliest activation sites in both benchmarks with deviations of only a fraction of the used spatial discretization size. Further, with the quality of the input data reduced by spatial undersampling and addition of noise, we demonstrate that an accurate identification of the sites of earliest activation is still feasible.

## Introduction

Computational models of cardiac function are increasingly considered as a clinical research tool with the perspective of being used, ultimately, as a diagnostic modality. Independently of which functional aspects are being considered, a key driving mechanism of cardiac electro–mechano–fluidic function is the sequence of electrical activation. Owing to its pivotal role, computer models intended for clinical applications must be parameterized in a patient-specific manner to approximate the electrical activation sequence in a given patient’s heart. Anatomical (Demoulin and Kulbertus 1972; Ono et al. 2009) as well as early experimental mapping studies (Durrer et al. 1970), using ex vivo human hearts provided evidence that electrical activation in the left ventricle (LV), i.e. the main pumping chamber that drives blood into the circulatory system, is initiated by the His–Purkinje system (Haissaguerre et al. 2016) at several specific sites of earliest activation (root points) which are located at the endocardial (inner) surface of the LV. In a first approximation it can be assumed that the healthy human LV is activated at these root points by a tri-fascicular conduction system (Rosenbaum et al. 1969) consisting of three major fascicles referred to as anterior, septal and posterior fascicle. Owing to the fast conduction properties of the Purkinje network tissue patches surrounding root points are activated fast enough so that their activation can be considered instantaneous. Size and location of these patches as well as the corresponding instants of their activation are key determinants shaping the activation sequence of the LV. Since the His–Purkinje system is highly variable in humans, there is significant interest in inverse methods for identifying these sites, ideally non-invasively.

In general, non-invasive electrocardiographic imaging attempts to reconstruct the spatio-temporal behavior of the electrical sources of the heart from electrocardiograms recorded from the body surface by solving the inverse problem of electrocardiography (Gulrajani et al. 1989). Solving this inverse problem is complicated by the non-uniqueness of the relation between myocardial sources and their signature outside the heart, recorded in the form of extracellular electrograms. The vast body of research found in the literature can be broadly categorized based on the regularization techniques used to rule out solutions that are unlikely on physiological grounds (Tikhonov and Arsenin 1977) and the model used for representing the cardiac sources, with the predominant source models being transmembrane voltage-based (He et al. 2003; Wang et al. 2010), extracellular-potential based (Rudy and Burnes 1999; Bear et al. 2018), and activation/recovery-based (van Dam et al. 2009; Erem et al. 2014; Han et al. 2015; Janssen et al. 2018). These models have their pros and cons in terms of verifiability with experimental data, the domains in which sources can be reconstructed—on epicardial and endocardial surfaces or transmurally throughout the myocardial wall—and their accuracy in pathological scenarios such as the presence of infarcts (Wang et al. 2013) or more complex non-physiological activation patterns such as arrhythmias (Rudy 2013). For a comprehensive overview of these aspects of ECG imaging we refer to the recent review of Cluitmans et al. (2018).

In this study we propose a novel method for identifying these sites of earliest activation from activation maps measured at the epicardial (outer) surface of the heart. Such maps can be obtained non-invasively from body surface potential maps within clinical routine using inverse mapping systems such as CardioInsight (Ramanathan et al. 2004). Epicardial activation maps depend not only on location and timing of initial activation sites, but also on the orthotropic conduction velocities within the LV wall. Therefore, in patient-specific applications, conduction velocity tensors have to be identified using fast forward computational models (Zettinig et al. 2014; Marchesseau et al. 2013a, b), or biophysically detailed models (Potse et al. 2014). The propagation of electrical wavefronts in the LV is modeled based on the viscous Eikonal equation which is able to represent activation sequences and takes into account the dependency of conduction velocity on wavefront curvature. Identification of sites of earliest activation is achieved by solving a minimization problem. Initially geometries are chosen which represent the activation sites. Then they are relocated based on a perturbation field until a minimal mismatch between the computed and the given activation maps at the epicardial surface is achieved. The perturbation field is designed to reduce the functional subject to minimization during the relocation process. The proposed method is tested in two numerical benchmarks, a generic 2D unit-square benchmark serving the sole purpose of theoretical analysis, and an anatomically accurate MRI-derived 3D human LV benchmark to demonstrate potential utility in a clinical context. For unperturbed input data, our localization method is able to accurately reconstruct earliest activation sites in both benchmarks with deviations of only a fraction of the used spatial discretization size. With the quality of the input data reduced by spatial undersampling and addition of noise, we demonstrate that an accurate identification is still feasible.

From a mathematical point of view the described problem can be interpreted as an inverse problem involving a non-linear elliptic PDE. On the activation sites $\omega _i$, $i=1,\ldots ,N$ an electrical depolarization wave is initiated which travels through the heart $\varOmega =U{\setminus } \cup _{i=1}^N\omega _i$. This is modelled by a nonlinear elliptic PDE, given by a viscous Eikonal equation, see Colli Franzone et al. (1990). The solution of the viscous Eikonal equation quantifies the arrival times of wave fronts at points in the heart $\varOmega$ or on its surface $\varGamma _O$. Since the wave is initiated on $\cup _{i=1}^N\omega _i$ the arrival time is zero on $\partial \cup _{i=1}^N\omega _i$ and thus the viscous Eikonal equation has zero Dirichlet boundary conditions on $\partial \cup _{i=1}^N\omega _i$ and Neumann boundary conditions on the rest of the boundary of $\varOmega$. Given measurements of the arrival times on the surface of the heart $\varGamma _O$ the positions of the activation sites $\omega _i$ are searched for. This inverse problem can be formulated as a shape optimization problem, see Delfour and Zolesio (2011) or Sokołowski and Zolésio (1992), in which the positions of $\omega _i$ is optimized such that the misfit between the measured data and the solution of the viscous Eikonal equation on $\varGamma _O$ is minimal. We assume that the shape and number of activation sites is known and stays constant during the optimization. Thus only the locations of the activation sites are changed during the optimization. For the derivation of the shape derivative of the shape functional we use a technique which does not require the shape differentiability of the geometry-to-state mapping, see Ito et al. (2008) and Laurain and Sturm (2016). In order to apply this technique we first prove the wellposedness of the state equation. It is a nonlinear elliptic PDE which can be transformed to a linear one using the Hopf–Cole transformation, see Capuzzo Dolcetta (2003). The proof of the continuous dependence of the state on the data requires non-standard techniques. Furthermore we prove the wellposedness of the linearized and adjoint state equation using the weak maximum principle. In order to compute the shape derivative the averaged adjoint technique from Laurain and Sturm (2016) is used. In this manner we arrive at domain-based representation of the shape derivative, in contrast to the more common boundary-based representation, see Sokołowski and Zolésio (1992). This simplifies the numerical implementation of the shape derivative in a finite element environment, since only domain integrals need to be calculated. For the calculation of the perturbation field which is the basis for changing the geometry of the activation sites in an iterative gradient based algorithm a linear elasticity problem is solved in which the shape derivative enters as righthand side. To give a brief account of the contents of the paper, in Sect. 2 after the statement of the model on which our approach is based, we give its mathematical analysis, involving primal, tangent, and adjoint equations, and the shape derivative. The use of this information for numerical realization is described in Sect. 3. Finally Sect. 4 contains the two benchmark examples alluded to above.

## Theoretical analysis

### Problem statement

Let $U\subset {\mathbb {R}}^d$, with $d=2$ or $d=3$, be a bounded domain with $C^{2,1}$ boundary, representing the cardiac domain. Within *U* we introduce *N* subdomains $\omega _i$ with $C^{2,1}$ boundaries $\partial \omega _i$, which represent the volumes of the earliest activation sites, also denoted as activation sources. The union of $\omega _i$ is denoted by $\omega =\cup _{i=1}^N\omega _i$ and its boundary by $\varGamma =\cup _{i=1}^N\partial \omega _i$. As such, $\varGamma$ is the surface from which activation spreads into our computational cardiac domain $\varOmega := U{\setminus }{\bar{\omega }}$. We have $\partial \varOmega =\varGamma \cup \partial U$, and thus $\varOmega$ is a bounded domain with $C^{2,1}$ boundary. In particular it is connected, but due to the holes it is not simply connected. Furthermore $\varGamma \subset \partial \varOmega$ is closed. We set $\varGamma _N=\partial U$, and further introduce the observatory boundary $\varGamma _O\subseteq \varGamma _N$, which in our application is given by epicardium of the heart. We consider the following minimization problem:

1 $$\begin{aligned} \min _{\varOmega ,\varGamma }J(\varOmega ,\varGamma )=\frac{1}{2}\int _{\varGamma _O}(T(x)-z(x))^2\mathrm {d}x \end{aligned}$$

subject to the viscous Eikonal equation in the form

2 $$\begin{aligned} \left\{ \begin{aligned} -\varepsilon \,{\text {div}}(M\nabla T)+|\nabla T|_M^2&=1&\quad \text {in}~\varOmega \\ T&=0&\quad \text {on}~\varGamma \\ -\varepsilon M\nabla T\cdot \underline{n}&=g&\quad ~\text {on}\,~\varGamma _N \end{aligned}\right. \end{aligned}$$

for some non-negative function *g*, and with

$$\begin{aligned} |\nabla T(x)|_M:=\sqrt{\nabla T(x)^*M(x)\nabla T(x)}. \end{aligned}$$

The function *T*(*x*) represents the activation time, while the epicardial activation input data is denoted by *z*(*x*) which is assumed to be an element of $L^\infty (\varGamma _O)$. The matrix *M*(*x*) models the squared cardiac conduction velocity (see Sect. 3.5). It is assumed to be symmetric and uniformly elliptic, i.e. there exists a $\alpha >0$ such that

$$\begin{aligned} M(x)\zeta \cdot \zeta \ge \alpha |\zeta |^2\quad \forall \zeta \in {\mathbb {R}}^d,~\forall x\in {\bar{U}}. \end{aligned}$$

For the rest of this work we use the notation $M\ge \alpha$. The vector $\underline{n}$ denotes the outer unit normal vector on $\varGamma _N$.

The use of Eikonal equations is well-established to approximate the excitation process in the myocardium. We refer, for instance, to Colli Franzone et al. (1990) where a careful singular perturbation technique analysis with respect to the thickness of the myocardial wall and the time taken by the excitation wave front to cross the heart wall is carried out on the basis of the bidomain equations to arrive at various forms of Eikonal equations (Colli Franzone et al. 1990, Section 5).

Problem (1) falls in the class of inverse shape problems. For the numerical solution of (1) we require the shape derivative of *J* with respect to $\varGamma$ in order to use it in a gradient decent method. As prerequisite we need to prove well-posedness of the state equation (2) which arises as PDE constraint in (1), and we analyze the tangent and adjoint equations.

### Well-posedness of the viscous Eikonal equation

In this section, we discuss the well-posedness of the equation

3 $$\begin{aligned} \left\{ \begin{aligned} -\,\varepsilon \,{\text {div}}(M \nabla T)+|\nabla T|_M^2&=f&\quad \text {in}~\varOmega \\ T&=0&\quad \text {on}~\varGamma \\ \varepsilon M\nabla T\cdot \underline{n}&=g&\quad \text {on}\,~\varGamma _N, \end{aligned}\right. \end{aligned}$$

for some functions *f*, *g* specified later. Using the transformation $T(x)=-\varepsilon \log (w(x)+1)$ this problem can be transformed into

4 $$\begin{aligned} \left\{ \begin{aligned} -\varepsilon ^2\,{\text {div}}(M \nabla w)+fw&=-f&\quad \text {in}~\varOmega \\ w&=0&\quad \text {on}~\varGamma \\ \varepsilon ^2M\nabla w\cdot \underline{n}+gw&=-g&\quad \text {on}\,~\varGamma _N, \end{aligned}\right. \end{aligned}$$

which is linear in the unknown *w*. Let us introduce the spaces

$$\begin{aligned} W^{1,p}_0(\varOmega \cup \varGamma _N):=\overline{{\mathcal {C}}_c^\infty (\varOmega \cup \varGamma _N)}^{W^{1,p}(\varOmega )}=\left\{ v\in W^{1,p}(\varOmega )|~v|_{\varGamma }=0\right\} \end{aligned}$$

for $1\le p<\infty$ which are equipped with the norm

$$\begin{aligned} \Vert v\Vert _{W^{1,p}_0(\varOmega \cup \varGamma _N)}:=\Vert \nabla v\Vert _{L^p(\varOmega )}. \end{aligned}$$

Moreover we set $V:=H^1_0(\varOmega \cup \varGamma _N):=W^{1,2}_0(\varOmega \cup \varGamma _N)$. For $p>1$ let $p'$ its conjugate exponent. We introduce the positive and negative part of *f* defined by $f^{+}:=\max (0,f)$ and $f^{-}:=\max (0,-f)$ as well as the embedding constant $c_p>0$ of the embedding $\Vert w\Vert _{L^{2p'}(\varOmega )}\le c_p\Vert w\Vert _V$. Next we require the following assumptions on the regularity of the data:

(Ai) $M\in {\mathcal {C}}^{0,\delta }({\bar{\varOmega }},{\mathbb {R}}^{d^2})$ with $0<\delta <1$, $M\ge \alpha /2$ and $\Vert M\Vert _{{\mathcal {C}}^{0,\delta }({\bar{\varOmega }},{\mathbb {R}}^{d^2})}\le \rho _M$
(Aii) $f\in L^p(\varOmega )$ with $\Vert f^{-}\Vert _{L^p(\varOmega )}\le \varepsilon ^2\alpha /4c_p^2$, $p>d$ and $\Vert f\Vert _{L^p(\varOmega )}\le \rho _f$
(Aiii) $g\in L^{\infty }(\varGamma _N)$ with $g\ge 0$ and $\Vert g\Vert _{L^\infty (\varGamma _N)}\le \rho _g$

#### Lemma 1

For every (*M*, *f*, *g*) satisfying (Ai), (Aii) and (Aiii), there exists a unique solution $w\in V$ of (4). Moreover the solution satisfies $w\in W^{1,p}_0(\varOmega \cup \varGamma _N)$ with $p>2$ if $d=2$, and with $p\in (3,6]$ if $d=3$, and

$$\begin{aligned} \Vert w\Vert _{W^{1,p}_0(\varOmega \cup \varGamma _N)}\le C \end{aligned}$$

where $C>0$ depends continuously on $\varepsilon$, $\alpha$, $\rho _M$, $\rho _f$ and $\rho _f$.

#### Proof

Let $\tau _N{:}\,V\rightarrow L^{2(d-1)/(d-2)}(\varGamma _N)$ denote the continuous trace operator onto $\varGamma _N$. Using the embedding $V\hookrightarrow L^{2d/(d-2)}(\varOmega )$ and (Aii), it is easy to see that the integral $\int _\varOmega fwv~\mathrm {d}x$ is well defined for every $v\in V$. Due to the mentioned properties of the trace operator $\tau _N$ and (Aiii) we can conclude that the boundary integral $\int _{\varGamma _N}gwv~\mathrm {d}s$ is well defined. Thus we can formulate the weak form of (4) as

5 $$\begin{aligned} \varepsilon ^2\int _\varOmega M\nabla w\cdot \nabla v~\mathrm {d}x+\int _\varOmega fwv~\mathrm {d}x+\int _{\varGamma _N}gwv~\mathrm {d}s=-\int _\varOmega fv~\mathrm {d}x-\int _{\varGamma _N}gv~\mathrm {d}s\nonumber \\ \end{aligned}$$

for all $v\in V$. To argue existence of a solution of (5) we use the Lax–Milgram theorem. To prove the required coercivity in *V* we estimate for any $w\in V$ using (Aii) and (Aiii)

$$\begin{aligned}&\varepsilon ^2\int _\varOmega M\nabla w\cdot \nabla w~\mathrm {d}x+\int _\varOmega (f^+-f^-)w^2~\mathrm {d}x+\int _{\varGamma _N}gw^2~\mathrm {d}s\\&\quad \ge \frac{\varepsilon ^2\alpha }{2}\Vert w\Vert _V^2-\Vert f^-\Vert _{L^p(\varOmega )}\Vert w\Vert _{L^{2p'}(\varOmega )}^2\\&\qquad \ge \left( \frac{\varepsilon ^2\alpha }{2}-c_p^2\Vert f^-\Vert _{L^{p}(\varOmega )}\right) \Vert w\Vert _V^2\ge \frac{\varepsilon ^2\alpha }{4}\Vert w\Vert _V^2. \end{aligned}$$

Thus we obtain coercivity and the existence of a unique solution *w* to (5). Moreover there exists a constant $C>0$ depending on $\alpha$ and $\varepsilon$ such that

$$\begin{aligned} \Vert w\Vert _V\le C(\Vert f\Vert _{L^p(\varOmega )}+\Vert g\Vert _{L^\infty (\varGamma _N)}). \end{aligned}$$

Next we argue additional regularity of *w*. For this purpose we consider the terms involving *fw* and *gw* as known inhomogeneities with $w\in V$. We show that the functionals $F_1(v):=\int _\varOmega fwv~\mathrm {d}x$ and $F_2(v):=\int _{\varGamma _N}gwv~\mathrm {d}s$ are elements of $(W^{1,p'}(\varOmega ))^*$ with $p'\in (1,2)$ for $d=2$ and $p'\in [6/5,3/2)$ for $d=3$. First we consider $F_1$. We recall the embedding $W^{1,p'}(\varOmega )\hookrightarrow L^{{\bar{q}}}(\varOmega )$ with ${\bar{q}}=dp'/(d-p')=dp/(dp-d-p)$ and ${\bar{q}}'=dp/(d+p)$. We prove that $fw\in L^{{\bar{q}}'}(\varOmega )$. Using Hölder’s inequality with $r=(d+p)/d$ resp. $r'=(d+p)/p$ we obtain

$$\begin{aligned} \Vert fw\Vert _{L^{{\bar{q}}'}(\varOmega )}\le \Vert f\Vert _{L^p(\varOmega )}\Vert w\Vert _{L^{d}(\varOmega )}\le c\Vert f\Vert _{L^p(\varOmega )}\Vert w\Vert _V \end{aligned}$$

and thus

$$\begin{aligned} \Vert F_1\Vert _{(W^{1,p'}(\varOmega ))^*}\le c\Vert f\Vert _{L^p(\varOmega )}\Vert w\Vert _V. \end{aligned}$$

Next we consider $F_2$. We recall from Adams and Fournier (2003, Theorem 5.22) that $\tau _N$ is continuous from $W^{1,p'}(\varOmega )$ to $L^q(\varGamma _N)$ with $q=(dp'-p')/(d-p')$. Next we verify that $g\tau _Nw\in L^{q'}(\varGamma _N)$ with $q'=p'(d-1)/d(p'-1)=p(d-1)/d$. We have

$$\begin{aligned} \Vert g\tau _Nw\Vert _{L^{{\bar{q}}'}(\varGamma _N)}\le \Vert g\Vert _{L^\infty (\varGamma _N)}\Vert \tau _Nw\Vert _{L^{{\bar{q}}'}(\varGamma _N)}\le c\Vert g\Vert _{L^\infty (\varGamma _N)}\Vert w\Vert _V \end{aligned}$$

since $\tau _N{:}\,V\rightarrow L^{2(d-1)/(d-2)}(\varGamma _N)$. Here the restriction $p\le 6$ is necessary. Then assumption (Aiii) implies the assertion. Finally we get

$$\begin{aligned} \Vert F_2\Vert _{(W^{1,p'}(\varOmega ))^*}\le c\Vert g\Vert _{L^\infty (\varGamma _N)}\Vert w\Vert _V. \end{aligned}$$

Moreover $v\mapsto \int _\varOmega fv~\mathrm {d}x$ and $v\mapsto \int _{\varGamma _N}g\tau _Nv~\mathrm {d}x$ are functionals from $(W^{1,p'}(\varOmega ))^*$. A functional *F* from $(W^{1,p'}(\varOmega ))^*$ can represented in the form

$$\begin{aligned} \langle F,v\rangle _{(W^{1,p'}(\varOmega ))^*,W^{1,p'}(\varOmega )}=\int _\varOmega f_1v+f_2\cdot \nabla v~\mathrm {d}x \end{aligned}$$

with $f_1\in L^{p}(\varOmega )$ and a vector field $f_2\in L^p(\varOmega ,{\mathbb {R}}^d)$, see Adams and Fournier (2003, Theorem 3.8). Thus the results from Troianiello (1987, Theorem 3.16) imply that $w\in W^{1,p}_0(\varOmega \cap \varGamma _N)$ holds and the existence of a constant *C* depending on $\rho _M$, $\varepsilon$ and $\alpha$ such that

$$\begin{aligned}&\Vert w\Vert _{W^{1,p}_0(\varOmega \cup \varGamma _N)}\le C \left( (\Vert g\Vert _{L^\infty (\varGamma _N)}+\Vert f\Vert _{L^p(\varOmega )})\Vert w\Vert _V\right. \\&\qquad \left. +\,\Vert g\Vert _{L^\infty (\varGamma _N)}+\Vert f\Vert _{L^p(\varOmega )}\right) \\&\quad \le C \left( (\Vert g\Vert _{L^\infty (\varGamma _N)}+\Vert f\Vert _{L^p(\varOmega )})^2+\Vert g\Vert _{L^\infty (\varGamma _N)}+\Vert f\Vert _{L^p(\varOmega )}\right) . \end{aligned}$$

These results are applicable since the Dirichlet part $\varGamma$ of $\partial \varOmega$ is closed. $\square$

In order to proof even higher regularity of *w* we use the following assumptions:

(Bi) $M\in {\mathcal {C}}^{1,\delta }({\bar{\varOmega }},{\mathbb {R}}^{d^2})$ with $M\ge \alpha$ and $\Vert M\Vert _{{\mathcal {C}}^{1,\delta }({\bar{\varOmega }},{\mathbb {R}}^{d^2})}\le \rho _M$
(Bii) $f\in {\mathcal {C}}^{0,\delta }({\bar{\varOmega }})$ with $f>0$ and $\Vert f\Vert _{{\mathcal {C}}^{0,\delta }({\bar{\varOmega }})}\le \rho _f$
(Biii) $g\in {\mathcal {C}}^{1,\delta }(\varGamma _N)$ with $g\ge 0$ and $\Vert g\Vert _{{\mathcal {C}}^{1,\delta }(\varGamma _N)}\le \rho _g$

for some $$0<\delta <1$$.

#### Lemma 2

Let Assumptions (Bi), (Bii) and (Biii) be satisfied. Then the solution of (4) satisfies $w\in {\mathcal {C}}^{2,\delta }({\bar{\varOmega }})$ with $0<\delta <1$ given according to the data. Moreover there exists a constant $C>0$ depending continuously on $\alpha$, $\varepsilon$, $\rho _M$, $\rho _f$ and $\rho _g$ such that

$$\begin{aligned} \Vert w\Vert _{{\mathcal {C}}^{2,\delta }({\bar{\varOmega }})}\le C \end{aligned}$$

and $-\,1< w(x)\le 0$ holds for all $x\in {\bar{\varOmega }}$.

#### Proof

Theorem 3.28 (ii) and 3.29 (ii) from Troianiello (1987) can be applied, since (4) can be written as

$$\begin{aligned} \left\{ \begin{aligned} -\varepsilon ^2\sum _{i,j=1}^dM_{i,j}\partial _{x_ix_j}w+\sum _{i=1}^da_i\partial _{x_i}w+fw&=-f&\quad \text {in}~\varOmega \\ w&=0&\quad \text {on}~\varGamma \\ \sum _{i=1}^db_i\partial _{x_i}w+gw&=-g&\quad \text {on}\,~\varGamma _N \end{aligned}\right. \end{aligned}$$

with $a_i:=-\varepsilon ^2\,{\text {div}}(M_i)\in {\mathcal {C}}^{0,\delta }({\bar{\varOmega }})$ ($M_i$ ith column of *M*) and $b_i:=\varepsilon ^2(Mn)_i\in {\mathcal {C}}^{1,\delta }(\varGamma _N)$ since $M\in {\mathcal {C}}^{1,\delta }({\bar{\varOmega }},{\mathbb {R}}^{d^2})$ and $\varGamma _N$ is of class ${\mathcal {C}}^{2,1}$. This gives us the stated regularity and the corresponding a priori estimate. Next we define $w^+:=\max (0,w)$ and $(w+1)^-:=\max (0,-(w+1))$. Since $(w+1)^-|_{\varGamma }=0$ we can test (5) with $v=-(w+1)^-$ and get

$$\begin{aligned} -\int _\varOmega f|(w+1)^-|^2~\mathrm {d}x= & {} -\varepsilon ^2\int _\varOmega M\nabla w\cdot \nabla (w+1)^-~\mathrm {d}x\\&\quad -\,\int _{\varGamma _N}g(w+1)(w+1)^-~\mathrm {d}s\\\le & {} \varepsilon ^2\int _\varOmega M\nabla (w+1)^-\cdot \nabla (w+1)^-~\mathrm {d}x\\&\quad +\,\int _{\varGamma _N}g|(w+1)^-|^2~\mathrm {d}s\ge 0 \end{aligned}$$

This implies $-\,1\le w$ in ${\bar{\varOmega }}$, since $f>0$. Testing (5) with $v=w^+$. We get

$$\begin{aligned} \int _\varOmega f|w^+|^2~\mathrm {d}x= & {} -\int _\varOmega fw^+~\mathrm {d}x -\varepsilon ^2\int _\varOmega M\nabla w\cdot \nabla w^+~\mathrm {d}x\\&-\,\int _{\varGamma _N}g(w+1)w^+~\mathrm {d}s\le 0. \end{aligned}$$

This implies $w\le 0$ in ${\bar{\varOmega }}$. Next we introduce the variable ${\hat{w}}=-(w+1)$ which satisfies the equation

$$\begin{aligned} \left\{ \begin{aligned} -\varepsilon ^2\,{\text {div}}(M \nabla {\hat{w}})+{\hat{w}}f&=0&\text {in}~\varOmega \\ {\hat{w}}&=-1&\text {on}~\varGamma \\ \varepsilon ^2M\nabla {\hat{w}}\cdot \underline{n}+{\hat{w}}g&=0&\text {on}\,~\varGamma _N. \end{aligned}\right. \end{aligned}$$

If the solution ${\hat{w}}$ were constant, it has to be equal to $-1$. However, in this case we have ${\hat{w}}=0$ in $\varOmega$, which is a contradiction. We define $O:=\max _{x\in {\bar{\varOmega }}}{\hat{w}}\in [-\,1,0]$, see above. First we assume $O=0$. Then Theorem 3.27 in Troianiello (1987) is applicable which states that such a maximum cannot be achieved on $\varOmega \cup \varGamma _N$. This is a contradiction. Thus $O\in [-\,1,0)$ and ${\hat{w}}\in [-\,1,0)$. This implies the assertion. $\square$

For the rest of this work we fix a $g\in {\mathcal {C}}^{1,\delta }(\varGamma _N)$ with $g\ge 0$, $0<\delta <1$ and $\Vert q\Vert _{{\mathcal {C}}^{1,\delta }(\varGamma _N)}\le \rho _g$. Let

$$\begin{aligned} Y=Y_M\times Y_f\subset C^{1,\delta }({\bar{\varOmega }},{\mathbb {R}}^{d^2})\times {\mathcal {C}}^{0,\delta }({\bar{\varOmega }}) \end{aligned}$$

be a reflexive Banach space which embeds compactly into ${\mathcal {C}}^{0,\delta }({\bar{\varOmega }},{\mathbb {R}}^{d^2})\times L^{p}(\varOmega )$ for some $0<\delta <1$, where the range of *p* is defined in Lemma 1. We define the set

6 $$\begin{aligned} B_Y:=\left\{ (M,f)\in Y{:}\,\Vert (M,f)\Vert _Y\le \rho ,~M\ge \alpha ,~f\ge \beta \right\} . \end{aligned}$$

for some $\rho =2\max (\rho _M,\rho _f),\beta >0$. Note that for $(M,f)\in B_Y$ conditions (Bi), (Bii) are satisfied.

#### Proposition 1

There exists a constant ${\bar{c}}\in (0,1)$ such that

$$\begin{aligned} -{\bar{c}}\le w(M,f;x)\le 0\quad \forall x\in {\bar{\varOmega }} \end{aligned}$$

for all $$(M,f)\in B_Y$$.

#### Proof

We shall employ a compactness argument. For this purpose we argue that $B_Y$ is compact in ${\mathcal {C}}^{0,\delta }({\bar{\varOmega }},{\mathbb {R}}^{d^2})\times L^{p}(\varOmega )$. The compact embedding of *Y* into ${\mathcal {C}}^{0,\delta }({\bar{\varOmega }},{\mathbb {R}}^{d^2})\times L^{p}(\varOmega )$ implies precompactness of $B_Y$. Moreover $B_Y$ is closed in ${\mathcal {C}}^{0,\delta }({\bar{\varOmega }},,{\mathbb {R}}^{d^2})\times L^{p}(\varOmega )$. Indeed, let $(M_n,f_n)_{n=1}^\infty \subset B_Y$ be a convergent sequence in ${\mathcal {C}}^{0,\delta }({\bar{\varOmega }},,{\mathbb {R}}^{d^2})\times L^{p}(\varOmega )$ with the limit point (*M*, *f*). It is easy to see that $M\ge \alpha$ holds. There exists a subsequence $(M_{n_k},f_{n_k})_{k=1}^\infty$ such that $f_{n_k}$ converges for almost every $x\in \varOmega$ to *f*. Thus *f* satisfies $f\ge \beta$. On another subsequence of this subsequence there holds $(M_{n_k},f_{n_k})\rightharpoonup (M,f)$ in *Y* due to the reflexivity of *Y*. Since $B_Y$ is convex and closed in *Y*, it is weakly closed in *Y*. Thus we have $(M,f)\in B_Y$ which implies the closedness of $B_Y$ in ${\mathcal {C}}^{0,\delta }({\bar{\varOmega }},,{\mathbb {R}}^{d^2})\times L^{p}(\varOmega )$. Finally this implies that $B_Y$ is a compact subset of ${\mathcal {C}}^{0,\delta }({\bar{\varOmega }},,{\mathbb {R}}^{d^2})\times L^{p}(\varOmega )$.

Next we define

$$\begin{aligned} B=\left\{ (M,f){:}\,\text {satisfy}~(Ai), (Aii)~\text {and}~\Vert (M,f)\Vert _{{\mathcal {C}}^{0,\delta }({\bar{\varOmega }},{\mathbb {R}}^{d^2})\times L^p(\varOmega )}\le K\right\} , \end{aligned}$$

where $K>\sup _{(M,f)\in B_Y} \Vert (M,f)\Vert _{C^{0,\delta }\times L^p(\varOmega )}$. We observe that there exists a $\kappa \in (0, \frac{\varepsilon ^2 \alpha }{8 c_p^2})$ such that for every $({\bar{M}},{\bar{f}}) \in B_Y$ the set

$$\begin{aligned} B_{\kappa }({\bar{M}}, {\bar{f}}):=\left\{ (M,f){:}\,\Vert (M-{\bar{M}},f-{\bar{f}})\Vert _{{\mathcal {C}}^{0,\delta }({\bar{\varOmega }},,{\mathbb {R}}^{d^2})\times L^p(\varOmega )}< \kappa \right\} \end{aligned}$$

satisfies the inclusion $B_{\kappa }({\bar{M}},{\bar{f}})\subset B$. For the coordinate *f* this is a consequence of the estimates

$$\begin{aligned} \Vert f^-\Vert _{L^{p}(\varOmega )} - \Vert f^+-{\bar{f}}\Vert _{L^{p}(\varOmega )}\le \Vert f-{\bar{f}}\Vert _{L^{p}(\varOmega )}\le \kappa , \end{aligned}$$

and hence

$$\begin{aligned} \Vert f^-\Vert _{L^{p}(\varOmega )} \le 2 \kappa < \frac{\varepsilon ^2 \alpha }{4 \,c_p^2}. \end{aligned}$$

We remark that Lemma 1 is applicable for $(M,f)\in B$ and thus for elements of $B_{\kappa }({\bar{M}},{\bar{f}})$ with $({\bar{M}},{\bar{f}})\in B_Y$. Next we choose an arbitrary $({\bar{M}},{\bar{f}})\in B_Y$ and $(M,f)\in B_{\kappa }({\bar{M}},{\bar{f}})$. Furthermore we introduce $(\delta M,\delta f)=({\bar{M}}-M,{\bar{f}}-f)$ and $\delta w={\bar{w}}-w=w({\bar{M}},{\bar{f}})-w(M,f)$. The solution *w* exists according to Lemma 1. The function $\delta w$ satisfies the equation

7 $$\begin{aligned}&\varepsilon ^2\int _\varOmega {\bar{M}}\nabla \delta w\cdot \nabla v~\mathrm {d}x+\int _\varOmega {\bar{f}}\delta wv~\mathrm {d}x+\int _{\varGamma _N}g\delta wv~\mathrm {d}s\nonumber \\&\quad =\varepsilon ^2\int _\varOmega \delta M\nabla w\cdot \nabla v~\mathrm {d}x-\int _\varOmega \delta f(w+1)v~\mathrm {d}x \end{aligned}$$

for all $v\in V$. Next we prove that $v\mapsto \varepsilon ^2\int _\varOmega \delta M\nabla w\cdot \nabla v~\mathrm {d}x$ is an element of $(W^{1,p'}(\varOmega ))^*$. Since $M\in {\mathcal {C}}^{0,\delta }({\bar{\varOmega }},{\mathbb {R}}^{d^2})$ and $w\in W^{1,p}_0(\varOmega \cup \varGamma _N)$, there holds

$$\begin{aligned} \varepsilon ^2\int _\varOmega \delta M\nabla w\cdot \nabla v~\mathrm {d}x\le \varepsilon ^2\Vert \delta M\Vert _{{\mathcal {C}}^{0,\delta }({\bar{\varOmega }},,{\mathbb {R}}^{d^2})}\Vert w\Vert _{W^{1,p}_0(\varOmega \cup \varGamma _N)}\Vert v\Vert _{W^{1,p'}(\varOmega )}. \end{aligned}$$

Then similar arguments as in the proof of Lemma 1 yield a constant $C>0$ depending on $\varepsilon$, $\alpha$ and $\rho _M$ such that

8 $$\begin{aligned}&\Vert \delta w\Vert _{W^{1,p}_0(\varOmega \cup \varGamma _N)}\le C\left( \left( \Vert {\bar{f}}\Vert _{L^p(\varOmega )}+\Vert g\Vert _{L^\infty (\varGamma _N)}\right) \Vert \delta w\Vert _V\right. \nonumber \\&\quad \left. +\,\varepsilon ^2\Vert \delta M\Vert _{{\mathcal {C}}^{0,\delta }({\bar{\varOmega }},{\mathbb {R}}^{d^2})}\Vert w\Vert _{W^{1,p}_0(\varOmega \cup \varGamma _N)}+\Vert \delta f\Vert _{L^p(\varOmega )}\Vert w+1\Vert _{W^{1,p}(\varOmega )}\right) \nonumber \\&\quad \le C\left( \left( \Vert {\bar{f}}\Vert _{L^p(\varOmega )}+\Vert g\Vert _{L^\infty (\varGamma _N)}\right) \right. \nonumber \\&\qquad \left( \varepsilon ^2\Vert \delta M\Vert _{{\mathcal {C}}^{0,\delta }({\bar{\varOmega }},{\mathbb {R}}^{d^2})}\Vert w\Vert _{W^{1,p}_0(\varOmega \cup \varGamma _N)}+\Vert \delta f\Vert _{L^p(\varOmega )}\Vert w+1\Vert _{W^{1,p}(\varOmega )}\right) \nonumber \\&\quad \left. +\,\varepsilon ^2\Vert \delta M\Vert _{{\mathcal {C}}^{0,\delta }({\bar{\varOmega }},{\mathbb {R}}^{d^2})}\Vert w\Vert _{W^{1,p}_0(\varOmega \cup \varGamma _N)}+\Vert \delta f\Vert _{L^p(\varOmega )}\Vert w+1\Vert _{W^{1,p}(\varOmega )}\right) , \end{aligned}$$

where *p* is specified in Lemma 1. The expressions involving *w* are estimated in terms of $\rho _g$, $\varepsilon$, $\alpha$ and *K*. Thus there holds

9 $$\begin{aligned} \Vert \delta w\Vert _{W^{1,p}_0(\varOmega \cup \varGamma _N)}\le h(\Vert \delta M\Vert _{{\mathcal {C}}^{0,\delta }({\bar{\varOmega }},{\mathbb {R}}^{d^2})},\Vert \delta f\Vert _{L^p(\varOmega )}), \end{aligned}$$

where $h{:}\,{\mathbb {R}}^2\rightarrow {\mathbb {R}}$ is a continuous function with $h(0,0)=0$.

Now, let $({\bar{M}}, {\bar{f}})$ be an arbitrary element in $B_Y$. By Lemma 2 there exists a constant ${\tilde{c}}={\tilde{c}}({\bar{M}},{\bar{f}})\in (0,1)$ such that $-{\tilde{c}}\le w({\bar{M}},{\bar{f}};x)\le 0$ for all $x\in {\bar{\varOmega }}$. Since $W^{1,p}_0(\varOmega \cup \varGamma _N)\hookrightarrow {\mathcal {C}}({\bar{\varOmega }})$ for $p>d$ and due to (9) there exists a $\gamma =\gamma ({\bar{M}},{\bar{f}})<\kappa$ such that

$$\begin{aligned} -\frac{1+{\tilde{c}}}{2}\le w(M,f;x)\quad \forall x\in {\bar{\varOmega }} \end{aligned}$$

for all $(M,f)\in B_{\gamma ({\bar{M}},{\bar{f}})}$. The family $\{B_{\gamma ({\bar{M}},{\bar{f}})}{:}\,({\bar{M}},{\bar{f}})\in B_Y\}$ is an open covering in ${\mathcal {C}}^{0,\delta }({\bar{\varOmega }},{\mathbb {R}}^{d^2})\times L^{p}(\varOmega )$ of the compact set $B_Y$. Hence there exists a finite subcover $\{B_{\gamma ({\bar{M}}_i,{\bar{f}}_i)}{:}\,({\bar{M}}_i,{\bar{f}}_i)\}_{i=1}^N$. Then we choose

$$\begin{aligned} {\bar{c}}:=\frac{1+\max _{1\le i\le N}{\tilde{c}}({\bar{M}}_i,{\bar{f}}_i)}{2}, \end{aligned}$$

to conclude the desired result. $\square$

With the help of Lemma 2 we are able to define $T=-\varepsilon \log (w+1)$ and calculate

$$\begin{aligned} \nabla T=-\frac{\varepsilon }{w+1}\nabla w,\quad {\text {div}}(M\nabla T)=-\frac{\varepsilon }{w+1}{\text {div}}(M\nabla w)+\frac{\varepsilon }{(w+1)^2}|\nabla w|_M^2. \end{aligned}$$

Thus there holds

$$\begin{aligned}&-\,\varepsilon \,{\text {div}}(M \nabla T)+|\nabla T|_M^2\\&\quad =\frac{\varepsilon ^2}{w+1}{\text {div}}(M\nabla w)-\frac{\varepsilon ^2}{(w+1)^2}|\nabla w|_M^2+\frac{\varepsilon ^2}{(w+1)^2}|\nabla w|_M^2=f. \end{aligned}$$

Moreover we have on the boundary

$$\begin{aligned} T|_{\varGamma }=-\varepsilon \log (1)=0,\quad \varepsilon M\nabla T\cdot \underline{n}|_{\varGamma _N}=\frac{-\varepsilon ^2}{w+1}M\nabla w\cdot \underline{n}|_{\varGamma _N} =g. \end{aligned}$$

We are now prepared to state the existence theorem for the state equation (3).

#### Theorem 1

Let $(M,f)\in B_Y$ where $B_Y$ is defined in (6). Then Eq. (3) has a unique solution $T\in {\mathcal {C}}^{2}({\bar{\varOmega }})$ satisfying

10 $$\begin{aligned} \Vert T\Vert _{{\mathcal {C}}^{2}({\bar{\varOmega }})}\le C_T, \end{aligned}$$

where $C_T$ only depends on $B_Y$.

#### Proof

Since existence of *T* was argued above only the estimate has to be proven. We know $T=-\varepsilon \log (w+1)$, $\nabla T=-\frac{\varepsilon }{w+1}\nabla w$ and

$$\begin{aligned} \partial _{x_ix_j}T=\frac{\varepsilon }{(w+1)^2}\partial _{x_i}w\partial _{x_j}w-\frac{\varepsilon }{w+1}\partial _{x_ix_j}w. \end{aligned}$$

Thus there holds

$$\begin{aligned} T(x)= & {} -\varepsilon \log (w(x)+1)\le -\varepsilon \log (-{\bar{c}}+1)\le K_1, \\ |\nabla T(x)|= & {} \varepsilon \frac{1}{(w+1)}|\nabla w(x)|\le \varepsilon \frac{1}{(-{\bar{c}}+1)}|\nabla w(x)|\le K_2 \end{aligned}$$

and

$$\begin{aligned} |D^2T(x)|\le \frac{\varepsilon }{(-{\bar{c}}+1)^2}|\nabla w(x)|+\frac{\varepsilon }{-{\bar{c}}+1}|D^2w(x)|\le K_3 \end{aligned}$$

where ${\bar{c}}$ is the constant from Proposition 1 and $K_i$ only depends on $B_Y$. This implies (10). $\square$

### Well-posedness of the tangent and adjoint equations

Let $T\in {\mathcal {C}}^2({\bar{\varOmega }})\cap V$ be the solution of the state equation for a $(M,f)\in B_Y$ and ${\hat{T}}$ for $({\hat{M}},{\hat{f}})\in B_Y$. Associated to the linearization of (2) we define the bilinear form $B{:}\,V\times V\rightarrow {\mathbb {R}}$ by

$$\begin{aligned} B(v,\varphi ):=\int _\varOmega \varepsilon M\nabla v\cdot \nabla \varphi +M\nabla (T+{\hat{T}})\cdot \nabla v\,\varphi ~\mathrm {d}x \end{aligned}$$

for any $\varphi ,v\in V$. Moreover we introduce the operators ${\mathcal {A}}{:}\,V\rightarrow V^*$ and ${\mathcal {A}}^*{:}\,V\rightarrow V^*$ defined by

$$\begin{aligned} \langle \mathcal {A}v,\varphi \rangle _{V^*,V}=B(v,\varphi )=\langle v,{\mathcal {A}}^*\varphi \rangle _{V,V^*} \end{aligned}$$

for all $$v,\varphi \in V$$.

#### Definition 1

For $F\in V^*$ we call $v\in V$ a solution of the linearized state equation if it solves the equation $\mathcal {A}v=F$ or equivalently

11 $$\begin{aligned} B(v,\varphi )=\langle F,\varphi \rangle _{V^*,V}\quad \forall \varphi \in V. \end{aligned}$$

#### Lemma 3

The mapping $(M,f)\mapsto T$ from $B_Y$ endowed with the topology of ${\mathcal {C}}^{0,\delta }({\bar{\varOmega }},{\mathbb {R}}^{d^2})\times L^6(\varOmega )$ to $W^{1,6}_0(\varOmega )$ is continuous.

#### Proof

Let *T* be the solution of the state equation for *M* and *f* and ${\tilde{T}}$ for ${\tilde{M}}$ and ${\tilde{f}}$. Let *w* be the solution of (4) for *M*, *f* and ${\tilde{w}}$ for ${\tilde{M}}$ and ${\tilde{f}}$. Due to Taylor expansion of 1 / *x* at ${\tilde{w}}(x)+1$ the partial derivative of the difference $\delta T:=T-{\tilde{T}}$ satisfies the equation

$$\begin{aligned}&\partial _{x_j}\delta T(x)=\frac{\varepsilon }{{\tilde{w}}(x)+1}\partial _{x_j}{\tilde{w}}(x)-\frac{\varepsilon }{w(x)+1}\partial _{x_j}w(x)\\&\quad =\left( \frac{\varepsilon }{{\tilde{w}}(x)+1}-\frac{\varepsilon }{w(x)+1}\right) \partial _{x_j}{\tilde{w}}(x)-\frac{\varepsilon }{w(x)+1}\partial _{x_j}\delta w(x)\\&\quad =\left( \frac{\varepsilon }{({\tilde{w}}(x)+1)^2}\delta w(x)-\frac{\varepsilon }{\eta (x)^3}\delta w(x)^2\right) \partial _{x_j}{\tilde{w}}(x)-\frac{\varepsilon }{w(x)+1}\partial _{x_j}\delta w(x), \end{aligned}$$

where $\delta w:=w-{\tilde{w}}$ and $\eta (x)$ lies between $w(x)+1$ and ${\tilde{w}}(x)+1$. Due to Proposition 1 we have

$$\begin{aligned}&|\partial _{x_j}\delta T(x)|\le \varepsilon |\delta w(x)|\left( \frac{1}{(-{\bar{c}}+1)^2}+\frac{{\bar{c}}}{(-{\bar{c}}+1)^3}\right) |\partial _{x_j}{\tilde{w}}(x)|\\&\quad +\,\frac{\varepsilon }{-{\bar{c}}+1}|\partial _{x_j}\delta w(x)|. \end{aligned}$$

Now estimate (9) for $\delta w$ in the proof of Proposition 1 with $p=6$ and Lemma 2 imply the assertion. $\square$

#### Proposition 2

Let $r\in (2,\infty )$ and $F\in W^{1,r'}(\varOmega )^*$. Then the linearized state equation has a unique solution $v\in W^{1,r}_0(\varOmega \cup \varGamma _N)$ and there exists a constant $C>0$ such that for all $(M,f)\in B_Y$

$$\begin{aligned} \Vert v\Vert _{W^{1,r}_0(\varOmega \cup \varGamma _N)}\le C(\Vert F\Vert _{W^{1,r'}(\varOmega )^*}). \end{aligned}$$

#### Proof

First we observe the following estimate

$$\begin{aligned}&\left| \int _\varOmega M\nabla (T+{\hat{T}})\cdot \nabla v\,v~\mathrm {d}x\right| \\&\quad \le \frac{\alpha \varepsilon }{2}\Vert v\Vert _V^2+\frac{1}{2\alpha \varepsilon }\Vert M\Vert ^2_{{\mathcal {C}}({\bar{\varOmega }},{\mathbb {R}}^{d^2})}\Vert \nabla (T+{\hat{T}})\Vert _{{\mathcal {C}}({\bar{\varOmega }})}^2\Vert v\Vert ^2_{L^2(\varOmega )}. \end{aligned}$$

Then we have

$$\begin{aligned}&B(v,v)+\lambda \Vert v\Vert ^2_{L^2(\varOmega )}\\&\quad \ge \frac{\alpha \varepsilon }{2}\Vert v\Vert _V^2+\left( \lambda -\frac{1}{2\alpha \varepsilon }\Vert M\Vert ^2_{{\mathcal {C}}({\bar{\varOmega }},{\mathbb {R}}^{d^2})}\Vert \nabla (T+{\hat{T}})\Vert _{{\mathcal {C}}({\bar{\varOmega }})}^2\right) \Vert v\Vert _{L^2(\varOmega )}^2\\&\qquad \ge \frac{\alpha \varepsilon }{2}\Vert v\Vert _V^2+(\lambda -\frac{1}{2\alpha \varepsilon }c^2\rho _M^2C_T^2)\Vert v\Vert _{L^2(\varOmega )}^2 \end{aligned}$$

for some $c>0$. Now for the choice $\lambda \ge \frac{1}{2\alpha \varepsilon }c^2\rho _M^2C_T^2$ the form *B* is coercive relative to $L^2(\varOmega )$. It can be easily checked that *B* is bounded. The bilinear form *B* is also defined on $H^1(\varOmega )\times V$ and there holds that $B(1,v)=0$ for any $v\in V$. Then Troianiello (1987, Theorem 2.4) implies that ${\mathcal {A}}$ satisfies the weak maximum principle. Thus the homogenous equation $Av=0$ has the unique solution 0. Then Troianiello (1987, Theorem 2.2) yields the existence of a unique solution $v\in V$ of $\mathcal {A}v=F$ for every $F\in V^*$ which satisfied the inequality

$$\begin{aligned} \Vert v\Vert _V\le \Vert {\mathcal {A}}^{-1}\Vert \Vert F\Vert _{V^*}. \end{aligned}$$

Next we discuss the dependence of $\Vert {\mathcal {A}}^{-1}\Vert$ on *M* and *T*. First we remark that *T* depends on *M* and *f*. Thus we prove that the mapping $(M,f)\mapsto {\mathcal {A}}$ is continuous from $B_Y$ endowed with the topology of ${\mathcal {C}}^{0,\delta }({\bar{\varOmega }},{\mathbb {R}}^{d^2})\times L^6(\varOmega )$ to ${\mathcal {L}}(V,V^*)$. Let (*M*, *f*) and $({\tilde{M}},{\tilde{f}})$ be elements of $B_Y$ and ${\mathcal {A}}$ resp. $\tilde{{\mathcal {A}}}$ the corresponding operators. Then we estimate

$$\begin{aligned}&\langle ({\mathcal {A}}-\tilde{{\mathcal {A}}})v,\varphi \rangle _{V^*,V}=\int _\varOmega (M-{\tilde{M}})\nabla v\cdot \nabla \varphi ~\mathrm {d}x+\int _\varOmega (M-{\tilde{M}})\nabla (T+{\hat{T}})\cdot \nabla v\varphi ~\mathrm {d}x\\&\qquad +\,2\int _\varOmega {\tilde{M}}\nabla (T-{\tilde{T}})\cdot \nabla v\varphi ~\mathrm {d}x\\&\quad \le c\left( \Vert M-{\tilde{M}}\Vert _{{\mathcal {C}}({\bar{\varOmega }},{\mathbb {R}}^{d^2})}(1+\Vert \nabla (T+{\hat{T}})\Vert _{L^6(\varOmega )})\right. \\&\qquad \left. +\,\Vert {\tilde{M}}\Vert _{{\mathcal {C}}({\bar{\varOmega }},{\mathbb {R}}^{d^2})}\Vert \nabla (T-{\tilde{T}})\Vert _{L^6(\varOmega )}\right) \Vert v\Vert _V\Vert \varphi \Vert _V \end{aligned}$$

Thus we have

$$\begin{aligned} \Vert {\mathcal {A}}-\tilde{{\mathcal {A}}}\Vert \le c \left( \Vert M-{\tilde{M}}\Vert _{{\mathcal {C}}({\bar{\varOmega }},{\mathbb {R}}^{d^2})}+\Vert \nabla (T-{\tilde{T}})\Vert _{L^6(\varOmega )}\right) , \end{aligned}$$

since $\Vert \nabla (T+{\hat{T}})\Vert _{L^6(\varOmega )}\le {\tilde{c}}\,C_T$ for some ${\tilde{c}}>0$ and $\Vert {\tilde{M}}\Vert _{L^\infty (\varOmega )}\le {\hat{c}}\rho _M$ for some ${\hat{c}}>0$. Then Lemma 3 implies the continuity of $(M,f)\mapsto {\mathcal {A}}$ from $B_Y\subset {\mathcal {C}}^{0,\delta }({\bar{\varOmega }},{\mathbb {R}}^{d^2})\times L^6(\varOmega )$ to ${\mathcal {L}}(V,V^*)$. Thus the mapping $(M,f)\mapsto {\mathcal {A}}^{-1}$ is continuous from $B_Y$ endowed with the topology of ${\mathcal {C}}^{0,\delta }({\bar{\varOmega }},{\mathbb {R}}^{d^2})\times L^6(\varOmega )$ to ${\mathcal {L}}(V^*,V)$. Since $B_Y$ is compact in ${\mathcal {C}}^{0,\delta }({\bar{\varOmega }},{\mathbb {R}}^{d^2})\times L^6(\varOmega )$ for some $0<\delta <1$ there exists a constant $C>0$ only depending on $B_Y$ such that $\Vert {\mathcal {A}}^{-1}\Vert \le C$.

Finally we apply Troianiello (1987, Theorem 3.16, (iv)) which implies that $v\in W^{1,r}_0(\varOmega \cup \varGamma _N)$ and

$$\begin{aligned} \Vert v\Vert _{W^{1,r}_0(\varOmega \cup \varGamma _N)}\le {\hat{C}}(\Vert F\Vert _{W^{1,r'}(\varOmega )^*}+\Vert v\Vert _V), \end{aligned}$$

where ${\hat{C}}$ depends on $\varepsilon$, $\alpha$, $\rho _M$ and $C_T$. $\square$

#### Definition 2

For $F\in V^*$ we call $\varphi \in V$ a solution of the adjoint state equation if it satisfies the equation ${\mathcal {A}}^*\varphi =F$ or equivalently

12 $$\begin{aligned} B(v,\varphi )=\langle F,v\rangle _{V^*,V}\quad \forall v\in V. \end{aligned}$$

#### Theorem 2

Let $r\in (2,\infty )$ and $F\in W^{1,r'}(\varOmega )^*$. Then Eq. (12) has a unique solution $\varphi \in W^{1,r}_0(\varOmega \cup \varGamma _N)$. Moreover there exists a constant $C>0$ such that for all $(M,f)\in B_Y$

13 $$\begin{aligned} \Vert \varphi \Vert _{W^{1,r}_0(\varOmega \cup \varGamma _N)}\le C(\Vert F\Vert _{W^{1,r'}(\varOmega )^*}). \end{aligned}$$

#### Proof

From the proof of Proposition 2 it follows that ${\mathcal {A}}{:}\,V\rightarrow V^*$ is continuous and bijective. Thus ${\mathcal {A}}^*{:}\,V\rightarrow V^*$ is also continuous and bijective. In particular we have $({\mathcal {A}}^*)^{-1}=({\mathcal {A}}^{-1})^*$. So the equation ${\mathcal {A}}^*\varphi =F$ has a unique solution $\varphi \in V$ for every $F\in V^*$ and

$$\begin{aligned} \Vert \varphi \Vert _V\le \Vert ({\mathcal {A}}^{-1})^*\Vert \Vert F\Vert _{V^*}=\Vert {\mathcal {A}}^{-1}\Vert \Vert F\Vert _{V^*}\le C\Vert F\Vert _{V^*} \end{aligned}$$

for some constant $C>0$ which is uniform in $(M,f)\in B_Y$. Then we apply Troianiello (1987, Theorem 3.16, (iv)) which implies that $\varphi \in W^{1,r}_0(\varOmega \cup \varGamma _N)$ and

$$\begin{aligned} \Vert \varphi \Vert _{W^{1,r}_0(\varOmega \cup \varGamma _N)}\le {\hat{C}}(\Vert F\Vert _{W^{1,r'}(\varOmega )^*}+\Vert \varphi \Vert _V), \end{aligned}$$

where ${\hat{C}}$ depends on $\varepsilon$, $\alpha$, $\rho _M$ and $C_T$. $\square$

Let us note that the strong form corresponding to (12) is formally given by

14 $$\begin{aligned} \left\{ \begin{aligned} -\varepsilon \,{\text {div}}(M\nabla \varphi )-{\text {div}}\left( M\nabla (T+{\hat{T}})\varphi \right)&=F|_{\varOmega }&\quad \text {in}~\varOmega \\ \varphi&=0&\quad \text {on}~\varGamma \\ \varepsilon M\nabla \varphi \cdot \underline{n}+2\varphi M\nabla T\cdot \underline{n}&=F|_{\varGamma _N}&\quad \,\text {on}~\varGamma _N. \end{aligned}\right. \end{aligned}$$

### Shape derivative of *J*

We follow the notation and strategy in Ito et al. (2008) and Laurain and Sturm (2016). For a field $h\in {\mathcal {C}}^{3}_c(U,{\mathbb {R}}^d)$ and $t>0$ we define the mappings $F_t{:}\,U\rightarrow {\mathbb {R}}^d$ by $F_t=\mathrm {id}_{{\mathbb {R}}^d}+th$. Then we introduce the perturbed domains $\varOmega _t=F_t(\varOmega )$ and the perturbed manifolds $\varGamma _t=F_t(\varGamma )$. Since *h* vanishes near $\varGamma _N$ there exists a $\tau >0$ such that $\varOmega _t\subset U$ for all $t\in [0,\tau ]$. Moreover, let $g\in {\mathcal {C}}^{2}(\varGamma _N)$ with $g\ge 0$ as well as $\Vert g\Vert _{{\mathcal {C}}^{1,\delta }(\varGamma _N)}\le \rho _g$ for some $0<\delta <1$ and $M\in {\mathcal {C}}^2({\bar{U}},{\mathbb {R}}^{d^2})$ with $M\ge \alpha$ be given. The perturbed state equation has the form

$$\begin{aligned} \int _{\varOmega _t} \varepsilon M\nabla T_t\cdot \nabla v+(M\nabla T_t\cdot \nabla T_t-1)v~\mathrm {d}x-\int _{\varGamma _N}gv~\mathrm {d}s=0\quad \forall v\in H^1_0(\varOmega _t\cup \varGamma _N), \end{aligned}$$

for $t\in [0,\tau ]$. We introduce

$$\begin{aligned} A(t)= & {} \xi (t)B^*(t)M(t)B(t), \text { where } B(t)=DF_t^{-*}, \quad \xi (t)=\det (DF_t),\\ M(t)= & {} M\circ F_t, \end{aligned}$$

and define the non-linear form $e{:}\,[0,\tau ]\times W^{1,4}_0(\varOmega \cup \varGamma _N)\times V\rightarrow {\mathbb {R}}$ as

$$\begin{aligned} e(t,T^t,v)=\int _\varOmega \varepsilon A(t)\nabla T^t\cdot \nabla v+(A(t)\nabla T^t\cdot \nabla T^t-\xi (t))v~\mathrm {d}x-\int _{\varGamma _N}gv~\mathrm {d}s. \end{aligned}$$

After transformation to the reference domain $\varOmega$, the perturbed state equation can be cast as

15 $$\begin{aligned} e(t,T^t,v)=0\quad \forall v\in V,\quad t\in [0,\tau ], \end{aligned}$$

with the relation between $T^t$ and $T_t$ given by $T^t=T_t \circ F_t$. Next we discuss the differentiability of *A*(*t*) and $\xi (t)$. We shall use the notation

$$\begin{aligned} M_vh=\left( \sum _{k=1}^dDM_kv_k\right) h, \end{aligned}$$

where $M_k$ stands for the kth column of *M*.

#### Lemma 4

There holds

$$\begin{aligned}&\lim _{t\downarrow 0}\frac{1}{t}\Vert \xi (t)-1-t\xi '(0)\Vert _{{\mathcal {C}}({\bar{\varOmega }})}=0, \\&\lim _{t\downarrow 0}\frac{1}{t}\Vert A(t)-M-tA'(0)\Vert _{{\mathcal {C}}({\bar{\varOmega }},{\mathbb {R}}^{d^2})}=0, \end{aligned}$$

where $$\xi '(0) = div (h)$$, and

16 $$\begin{aligned} A'(0)v={\text {div}}(h)Mv-DhMv+M_vh-MDh^*v,\quad \text {for}\,\,v\in {\mathbb {R}}^d. \end{aligned}$$

#### Proof

Let $x\in {\bar{\varOmega }}$ be arbitrary. The function $\xi (t;x)$ has the form

17 $$\begin{aligned} \xi (t;x)=1+{\text {tr}}(Dh(x))t-\det (Dh(x))t^2,\quad d=2 \end{aligned}$$

and

$$\begin{aligned} \xi (t;x)= & {} 1+{\text {tr}}(Dh(x))t-(\det (Dh_1(x))+\det (Dh_2(x))+\det (Dh_3(x)))t^2\\&\quad +\,\det (Dh(x))t^3,\quad d=3 \end{aligned}$$

where $Dh_i$ are the principal minors of *Dh*. Thus we have

$$\begin{aligned} \frac{1}{t}|\xi (t;x)-1-t\,{\text {div}}(h(x))|\le 3\Vert Dh\Vert _{{\mathcal {C}}({\bar{\varOmega }},{\mathbb {R}}^{d^2})}^2t+\Vert Dh\Vert _{{\mathcal {C}}({\bar{\varOmega }},{\mathbb {R}}^{d^2})}^3t^2. \end{aligned}$$

Thus the first assertion is proven. Let us turn to the differentiability of $t\mapsto A(t)$. Since $M\in {\mathcal {C}}^2({\bar{U}},{\mathbb {R}}^{d^2})$ and ${\bar{U}}$ is compact it follows that $t \mapsto M(x+th(x))$ is differentiable from $[0, \infty )$ to ${\mathcal {C}}({\bar{U}},{\mathbb {R}}^{d^2})$ at $t=0^+$. The derivative can be conveniently computed by its action on any $v\in {\mathbb {R}}^d$

$$\begin{aligned} \partial _tM(t)v|_{t=0}=\sum _{k=1}^d\partial _t M_k(t)t|_{t=0}v_k=\sum _{k=1}^dDM_khv_k=\left( \sum _{k=1}^dDM_kv_k\right) h. \end{aligned}$$

Now let $x\in {\bar{\varOmega }}$ be arbitrary and let *t* be so small such that $t\Vert Dh^*\Vert _{{\mathcal {C}}({\bar{\varOmega }},{\mathbb {R}}^{d^2})}<1$. Then there holds

$$\begin{aligned}&\frac{1}{t}\Vert B(t;x)-\mathrm {Id}+tDh(x)^*\Vert =\frac{1}{t}\left\| \sum _{k=0}^{\infty }(-t)^k(Dh(x)^*)^k-\mathrm {Id}+tDh(x)^*\right\| \\&\quad \le \sum _{k=2}^{\infty }t^{k-1}\Vert Dh^*\Vert _{{\mathcal {C}}({\bar{\varOmega }},{\mathbb {R}}^{d^2})}^k \end{aligned}$$

A similar proof shows

$$\begin{aligned} \lim _{t\downarrow 0}\frac{1}{t}\Vert B^*(t)-\mathrm {Id}+tDh\Vert _{{\mathcal {C}}({\bar{\varOmega }},{\mathbb {R}}^{d^2})}=0. \end{aligned}$$

Utilizing the product rule on $A(t)=\xi (t)B^*(t)M(t)B(t)$ leads us to (16). $\square$

The formulas for $\xi$ and *A* also provide the following result.

#### Lemma 5

The mappings $t\mapsto A(t)$ from $[0,\tau ]$ to ${\mathcal {C}}^1({\bar{\varOmega }},{\mathbb {R}}^{d^2})$ and $t\mapsto \xi (t)$ from $[0,\tau ]$ to ${\mathcal {C}}^1({\bar{\varOmega }})$ are continuous in 0.

Let $Y=Y_M\times Y_f=W^{2,s}(\varOmega ,{\mathbb {R}}^{d^2})\times W^{1,s}(\varOmega )\subset {\mathcal {C}}^{1,\delta }({\bar{\varOmega }},{\mathbb {R}}^{d^2})\times {\mathcal {C}}^{0,\delta }({\bar{\varOmega }})$ with $s>d$ and $\delta = 1-d/s$. Then *Y* is compactly embedded in $C^{0,\delta }({\bar{\varOmega }},{\mathbb {R}}^{d^2})\times L^{p}(\varOmega )$ for any $0<\delta <1$ and $p>d$. Due to the last lemma there exists a $\tau$ such that $A(t)\ge \alpha /2$ and $\xi (t)\ge 1/2$ for all $t\in [0,\tau ]$. Furthermore there exists a $\rho >0$ such that $\Vert (A(t),\xi (t))\Vert _Y\le \rho$ for all $t\in [0,\tau ]$ holds. Then we define the set

$$\begin{aligned} B_Y=\{(M,f)\in Y{:}\,\Vert (M,f)\Vert _Y\le \rho ,~M\ge \alpha /2, f\ge 1/2\} \end{aligned}$$

and get

$$\begin{aligned} \{(A(t),\xi (t)){:}\,t\in [0,\tau ]\}\subset B_Y. \end{aligned}$$

Thus we have:

#### Proposition 3

The perturbed state equation has a unique solution $T^t\in {\mathcal {C}}^2({\bar{\varOmega }})\cap V\hookrightarrow W^{1,4}_0(\varOmega \cup \varGamma _N)$.

#### Proof

This follows directly from Theorem 1. $\square$

The perturbed cost functional can be written as

18 $$\begin{aligned} J(\varOmega _t,\varGamma _t)=j(t,T^t)=\frac{1}{2}\int _{\varGamma _O}(T^t-z)^2\mathrm {d}x \end{aligned}$$

subject to $e(t,T^t,v)=0$ for all $v\in V$. Next we characterize the shape derivative

$$\begin{aligned} dJ(\varOmega ,\varGamma )h=\lim _{t\downarrow 0}\frac{J(\varOmega _t,\varGamma _t)-J(\varOmega ,\varGamma )}{t} \end{aligned}$$

at $\varOmega$ in direction *h*. For this purpose we define the Lagrange functional

$$\begin{aligned} L(t,T^t,p)=j(t,T^t)+e(t,T^t,p) \end{aligned}$$

for some $p\in V$ and $t\in [0,\tau ]$. We shall follow Laurain and Sturm (2016) to show that

19 $$\begin{aligned} dJ(\varOmega ,\varGamma )h=\frac{\mathrm {d}}{\mathrm {d}t} L(t,T^t,\varphi ^t)|_{t=0}, \end{aligned}$$

where $T^t$ solves (15) and $\varphi ^t$ solves the averaged adjoint equation

20 $$\begin{aligned} \int _0^1d_{T}L(t,sT^t+(1-s)T^0,\varphi ^t)\delta T~\mathrm {d}s=0\quad \forall \delta T\in W^{1,4}_0(\varOmega \cup \varGamma _N). \end{aligned}$$

At first we characterize the right hand side of (19). First we observe that

$$\begin{aligned} d_{T}L(t,T^t,\varphi ^t)\delta T= & {} \int _{\varGamma _O}(T^t-z)\delta T~\mathrm {d}s\\&+\,\int _\varOmega \varepsilon A(t)\nabla \delta T\cdot \nabla \varphi ^t+2A(t)\nabla T^t\cdot \nabla \delta T\varphi ^t~\mathrm {d}x. \end{aligned}$$

Since $T^t$ and $T^0$ appear linearly in (20), the averaged adjoint equation amounts to

21 $$\begin{aligned}&\int _{\varGamma _O}([T^t]-z)\delta T~\mathrm {d}s+\int _\varOmega \varepsilon A(t)\nabla \delta T\cdot \nabla \varphi ^t+2A(t)\nabla [T^t]\cdot \nabla \delta T\varphi ^t~\mathrm {d}x=0\nonumber \\&\quad \forall \delta T\in W^{1,4}_0(\varOmega \cup \varGamma _N), \end{aligned}$$

where $$[T^t]=1/2(T^t+T^0)\in {\mathcal {C}}^2({\bar{\varOmega }})$$.

#### Proposition 4

The averaged adjoint equation has a unique solution $\varphi ^t\in W^{1,r}_0(\varOmega \cup \varGamma _N)$ with $r\in (d,\infty )$.

#### Proof

We need to prove that $v\mapsto \int _{\varGamma _O}([T^t]-z)\tau _Nv~\mathrm {d}s$ is an element of $W^{1,r'}(\varOmega )^*$. We know that $\tau _N$ is continuous from $W^{1,r'}(\varOmega )$ to $L^q(\varGamma _O)$ with $q=(dr'-r')/(d-r')$. Thus we need to show that $[T^t]|_{\varGamma _O}-z\in L^{q'}(\varGamma _O)$ with $q'=r'(d-1)/d(r'-1)=r(d-1)/d$. This is true since $T\in {\mathcal {C}}^2({\bar{\varOmega }})$ and $z\in L^{\infty }(\varGamma _O)$. $\square$

In order to justify (19) we need the following technical lemma.

#### Lemma 6

Further let $T^t$ and $\varphi ^t$ be the solutions of (15) and of (20) for $t\in (0,\tau ]$. Then we have

$$\begin{aligned} T^t&\rightarrow T^{0}\quad \text {in}~W^{1,6}_0(\varOmega \cup \varGamma _N)~\text {for}~t\downarrow 0,\\ \varphi ^t&\rightarrow \varphi ^0\quad \text {in}~V~\text {for}~t\downarrow 0. \end{aligned}$$

#### Proof

The first result follows from Lemmas 3 and 5. Let $\varphi ^t$ be the solution of the averaged adjoint state equation (21) for *A*(*t*), $[T^t]=1/2(T^t+T^0)$ and *z*. We define $\delta \varphi =\varphi ^t-\varphi ^0$ which solves

$$\begin{aligned}&\int _\varOmega \varepsilon A(t)\nabla v\cdot \nabla \delta \varphi +A(t)\nabla (T^t+T^0)\nabla v\delta \varphi ~\mathrm {d}x\\&\quad =\int _\varOmega \varepsilon (M-A(t))\nabla v\cdot \nabla \varphi ^0+(2(M-A(t))\nabla T^0-A(t)\nabla \delta T)\cdot \nabla v\varphi ^0~\mathrm {d}x\\&\qquad +\,\frac{1}{2}\int _{\varGamma _O}\delta Tv~\mathrm {d}s \end{aligned}$$

for all $v\in V$. Next we show that $v\mapsto \int _\varOmega (M-A(t))\nabla T^0\nabla v\varphi ^0~\mathrm {d}x$ is an element of $V^*$. This follows from the fact that $\varphi ^0\in W^{1,r}_0(\varOmega \cup \varGamma _N)\hookrightarrow {\mathcal {C}}({\bar{\varOmega }})$ and $\nabla T^0\in {\mathcal {C}}^1({\bar{\varOmega }},{\mathbb {R}}^d)$. Moreover the functional $v\mapsto \int _\varOmega A(t)\nabla \delta T\nabla v\varphi ^0~\mathrm {d}x$ is also a functional in $V^*$, since $\delta T\in W^{1,6}_0(\varOmega \cup \varGamma _N)$. According to the proof of Theorem 2 there holds

$$\begin{aligned}&\Vert \delta \varphi \Vert _{V}\le C\left( \varepsilon \Vert A(t)-M\Vert _{{\mathcal {C}}({\bar{\varOmega }},{\mathbb {R}}^{d^2})}\Vert \varphi ^0\Vert _{W^{1,r}_0(\varOmega \cup \varGamma _N)}\right. \\&\quad +\,\Vert \varphi ^0\Vert _{W^{1,r}_0(\varOmega \cup \varGamma _N)}\Vert A(t)-M\Vert _{{\mathcal {C}}({\bar{\varOmega }},{\mathbb {R}}^{d^2})}\Vert T^0\Vert _{{\mathcal {C}}^2({\bar{\varOmega }})}\\&\quad \left. +\,\Vert A(t)\Vert _{{\mathcal {C}}({\bar{\varOmega }},{\mathbb {R}}^{d^2})}\Vert \varphi ^0\Vert _{W^{1,r}_0(\varOmega \cup \varGamma _N)}\Vert \delta T\Vert _{W^{1,6}_0(\varOmega \cup \varGamma _N)}+\Vert \delta T\Vert _{W^{1,6}_0(\varOmega \cup \varGamma _N)}\right) , \end{aligned}$$

with $C>0$ independent of *t*. Moreover due to Theorem 2 there exists a constant $c_1>0$ depending only on $B_Y$ such that $\Vert \varphi ^0\Vert _{W^{1,r}_0(\varOmega \cup \varGamma _N)}<c_1$ holds. Furthermore there holds $\Vert T^0\Vert _{{\mathcal {C}}^2({\bar{\varOmega }})}\le C_T$ and $\Vert A(t)\Vert _{{L^\infty }(\varOmega )}\le c_2\rho _M$ with $c_2$ independent of *t*. This finishes the proof using Lemma 5. $\square$

We introduce the outer product $v\otimes w=vw^*$ for $v,w\in {\mathbb {R}}^d$ and the inner product $G{:}\,N=\text {trace}(GN^*)$ for $G,N\in {\mathbb {R}}^{d\times d}$. Now we have all necessary ingredients to prove the main result of this subsection.

#### Theorem 3

The shape derivative $dJ(\varOmega ,\varGamma )$ of *J* defined in (18) satisfies

22 $$\begin{aligned} { DJ}(\varOmega ,\varGamma )h=\frac{\mathrm {d}}{\mathrm {d}t} L(t,T^t,\varphi ^t)|_{t=0}= \int _{\varOmega }S_1{:}\,Dh+S_0\cdot h~\mathrm {d}x \end{aligned}$$

for any $h\in {\mathcal {C}}_c^3(U,{\mathbb {R}}^d)$, where $S_i$, $i=0,1$ have the form

23 $$\begin{aligned} S_1&=\mathrm {Id}_{{\mathbb {R}}^d}(\varepsilon M\nabla T\cdot \nabla \varphi +(|\nabla T|_M^2-1)\varphi )-\varepsilon (\nabla T\otimes M\nabla \varphi +\nabla \varphi \otimes M\nabla T)\nonumber \\&\quad -2\nabla T\otimes M\nabla T\varphi , \end{aligned}$$

24 $$\begin{aligned} S_0&=\varepsilon M_{\nabla T}^*\nabla \varphi +M_{\nabla T}^*\nabla T\varphi . \end{aligned}$$

#### Proof

We apply Theorem 2.1 from Laurain and Sturm (2016). Thus we need to prove that

$$\begin{aligned} \lim _{t\downarrow 0}\frac{1}{t}(L(t,T^0,\varphi ^t)-L(0,T^0,\varphi ^t))=\partial _tL(0,T^0,\varphi ^0). \end{aligned}$$

The functional *J* only depends on *t* through $T^t$. Thus we have

$$\begin{aligned}&\left| \frac{1}{t}(L(t,T^0,\varphi ^t)-L(0,T^0,\varphi ^t))-\partial _tL(0,T^0,\varphi ^0)\right| \\&\quad =\left| \frac{1}{t}(e(t,T^0,\varphi ^t)-e(0,T^0,\varphi ^t))-\partial _te(0,T^0,\varphi ^0)\right| \\&\quad =\frac{1}{t}\left| \int _\varOmega \varepsilon (A(t)-M-tA'(0))\nabla T^0\cdot \nabla \varphi ^t+tA'(0)\nabla T^0\cdot \nabla (\varphi ^t-\varphi ^0)\right. \\&\qquad \left. +\,(A(t)-M-tA'(0))\nabla T^0\cdot \nabla T^0\varphi ^t+tA'(0)\nabla T^0\cdot \nabla T^0(\varphi ^t-\varphi ^0)\right. \\&\qquad \left. -\,(\xi (t)-1-t\xi '(0))\varphi ^t-t\xi '(0)(\varphi ^t-\varphi ^0)~\mathrm {d}x\right| \\ \end{aligned}$$

Thus we can estimate in the following way:

$$\begin{aligned}&\left| \frac{1}{t}(L(t,T^0,\varphi ^t)-L(0,T^0,\varphi ^t))-\partial _tL(0,T^0,\varphi ^0)\right| \\&\quad \le \frac{\varepsilon }{t}\Vert A(t)-M-tA'(0)\Vert _{{\mathcal {C}}({\bar{\varOmega }},{\mathbb {R}}^{d^2})}\Vert T^0\Vert _{V}\Vert \varphi ^t\Vert _{V}\\&\qquad +\,\varepsilon \Vert A'(0)\Vert _{{\mathcal {C}}({\bar{\varOmega }},{\mathbb {R}}^{d^2})}\Vert T^0\Vert _{V}\Vert \varphi ^t-\varphi ^0\Vert _{V}\\&\qquad +\,c\left( \frac{1}{t}\Vert A(t)-M-tA'(0)\Vert _{{\mathcal {C}}({\bar{\varOmega }},{\mathbb {R}}^{d^2})}\Vert \nabla T^0\Vert _{{\mathcal {C}}({\bar{\varOmega }},{\mathbb {R}}^d)}^2\Vert \varphi ^t\Vert _{V}\right. \\&\qquad \left. +\,\Vert A'(0)\Vert _{{\mathcal {C}}({\bar{\varOmega }},{\mathbb {R}}^{d^2})}\Vert \nabla T^0\Vert _{{\mathcal {C}}({\bar{\varOmega }},{\mathbb {R}}^d)}^2\Vert \varphi ^t-\varphi ^0\Vert _{V}\right) \\&\qquad +\,{\tilde{c}}\left( \frac{1}{t}\Vert \xi (t)-1-t\xi '(0)\Vert _{{\mathcal {C}}({\bar{\varOmega }})}\Vert \varphi ^t\Vert _{V}+\Vert \xi '(0)\Vert _{{\mathcal {C}}({\bar{\varOmega }})}\Vert \varphi ^t-\varphi ^0\Vert _{V}\right) . \end{aligned}$$

Then Lemmas 6 and 4 imply the assertion. In order to calculate

$$\begin{aligned} \frac{\mathrm {d}}{\mathrm {d}t} L(t,T^t,\varphi ^t)|_{t=0} \end{aligned}$$

we recall Lemma 4 and in particular (16). We obtain

$$\begin{aligned} \frac{\mathrm {d}}{\mathrm {d}t} L(t,T^t,\varphi ^t)|_{t=0}=\int _{\varOmega }\varepsilon A'(0)\nabla T^0\cdot \nabla \varphi ^0+(A'(0)\nabla T^0\cdot \nabla T^0-{\text {div}}(h))\varphi ^0~\mathrm {d}x \end{aligned}$$

with

$$\begin{aligned} A'(0)v={\text {div}}(h)Mv-DhMv+M_vh-MDh^*v,\quad \text {for}\,v\in {\mathbb {R}}^d. \end{aligned}$$

Next we give a more usable formula for the shape derivative. For convenience we suppress the superscript for $T^0$ and $\varphi ^0$ in the following. In particular we have

$$\begin{aligned} \varepsilon A'(0)\nabla T\cdot \nabla \varphi&=(\varepsilon M\nabla T\cdot \nabla \varphi )\,\mathrm {Id}_{{\mathbb {R}}^d}{:}\,Dh\\&\quad -\,(\varepsilon \nabla \varphi \otimes M\nabla T){:}\,Dh+(\varepsilon M_{\nabla T}^*\nabla \varphi )\cdot h\\&\quad -\,(\varepsilon \nabla T\otimes M\nabla \varphi ){:}\,Dh,\\ A'(0)\nabla T\cdot \nabla T\varphi&=\varphi |T|_M^2\mathrm {Id}_{{\mathbb {R}}^d}{:}\,Dh-(\nabla T\otimes M\nabla T\varphi ){:}\,Dh+(M_{\nabla T}^*\nabla T\varphi )\cdot h\\&\quad -\,(\nabla T\otimes M\nabla T\varphi ){:}\,Dh,\\ {\text {div}}(h)\varphi&=\varphi \,\mathrm {Id}_{{\mathbb {R}}^d}{:}\,Dh. \end{aligned}$$

$$\square $$

## Practical implementation

In this section we describe the practical implementation of an algorithm utilizing the shape derivative *DJ* for the reconstruction of the locations of the activation sites. We assume that these sites have the form $\omega _i=B_{r_i}(x_i)$ with radii $r_i$ and midpoints $x_i$, $i=1,\ldots ,N$. For these activation sites we reconstruct the midpoints $x_i$.

### The state and adjoint state equations

Since the state equation is of nonlinear elliptic type which in practically relevant situations is posed on domains with challenging geometry, we propose to solve it using linear finite elements and a Newton method. For convenience we recall the state equation as

25 $$\begin{aligned} e(T,v)= & {} \int _{\varOmega }\varepsilon M\nabla T\cdot \nabla v+(|\nabla T|_M^2-1)v~\mathrm {d}x-\int _{\varGamma _N}g_2v~\mathrm {d}s=0\nonumber \\&\quad \forall v\in W^{1,4}_0(\varOmega \cup \varGamma _N). \end{aligned}$$

In order to set up a Newton method we need to calculate the derivative of *e*, in particular we have

26 $$\begin{aligned} d_T e(T,\varphi )v = \int _{\varOmega }\varepsilon M\nabla v\cdot \nabla \varphi +2M\nabla T\cdot \nabla v\varphi ~\mathrm {d}x. \end{aligned}$$

The Newton equation is well posed, see Proposition 2. For a given solution *T* of the state equation, the adjoint state equation in the variable $\varphi \in V$ has the form

27 $$\begin{aligned} d_Te(T,\varphi )v= & {} \int _{\varOmega }\varepsilon M\nabla v\cdot \nabla \varphi +2M\nabla T\cdot \nabla v \varphi \,\mathrm {d}x \nonumber \\&+\,\int _{\varGamma _N}(T-z)v~\mathrm {d}x=0,\quad \forall v\in V. \end{aligned}$$

This is a linear elliptic equation of convection-diffusion type, which we again solve by linear finite elements.

### Domain perturbation

While the overall source localization algorithm requires only a displacement of the current source locations, we still calculate a vector field for the perturbation over the whole domain ${\bar{\varOmega }}$. This vector field *h* is chosen as the solution of the vector valued elliptic equation

28 $$\begin{aligned} \int _U Dh {:}\,Dv+h\cdot v~\mathrm {d}x=-\int _\varOmega S_1 {:}\,Dv+S_0\cdot v~\mathrm {d}x, \quad \forall v\in H^1_{0}(U,{\mathbb {R}}^d), \end{aligned}$$

where $S_i$, $i=0,1$ are defined in (24) resp. (23). We remark that *h* is defined on *U* and not only on $\varOmega$. The last equation is solved using linear finite elements. We also note that

$$\begin{aligned} { DJ}(\varOmega ,\varGamma )h=-\int _U Dh{:}\,Dh+h\cdot h~\mathrm {d}x\le 0, \end{aligned}$$

and thus *h* is a decent direction for *J*. Since we are only interested in the shift of the midpoints $x_i$ of the balls $\omega _i$, we average *h* over $\omega _i$, $i=i,\ldots ,N$, in order to get a shift of the midpoints.

### Finite element solver implementation

The domain $\varOmega$ is discretized using tetrahedral elements and linear Ansatz functions $\{\psi _i\}$. As such, there are three linear systems to be solved at least once in each iteration of the source localization loop:

1. The linear equation in the Newton iteration $K_N \, \underline{T} = \underline{f}_N$, with
  $$\begin{aligned} K_N ^{i,j}= & {} \int _{\varOmega } \varepsilon M \nabla \psi _i \cdot \nabla \psi _j + 2 \, (M \nabla T \cdot \nabla \psi _i) \, \psi _j \, \mathrm {d}x \\ \underline{f}_N^{i}= & {} - \int _{\varOmega } \varepsilon M \nabla T \cdot \nabla \psi _i + (M \nabla T \cdot \nabla T - 1) \, \psi _i \, \mathrm {d}x. \end{aligned}$$
2. The adjoint state equation $K_A \, \underline{\varphi } = \underline{f}_A$, with
  $$\begin{aligned} K_A ^{i,j}= & {} \int _{\varOmega } \varepsilon M \nabla \psi _j \cdot \nabla \psi _i + 2 \, (M \nabla T \cdot \nabla \psi _j) \, \psi _i \, \mathrm {d}x \\ \underline{f}_A^{i}= & {} \int _{\varGamma _N} (T - z) \, \psi _i \, \mathrm {d}x. \end{aligned}$$
3. The domain perturbation equation $K_S \, \underline{h} = \underline{f}_S$, with
  $$\begin{aligned} K_S ^{i,j}= & {} I_{3 \times 3} \, \int _{\varOmega } \delta _x \psi _i \, \delta _x \psi _j + \delta _y \psi _i \, \delta _y \psi _j + \delta _z \psi _i \, \delta _z \psi _j + \psi _i \, \psi _j \, \mathrm {d}x \\ \underline{f}_S^{i,1}= & {} \int _{\varOmega } S_1^{1,1}\delta _x \psi _i + S_1^{1,2}\delta _y \psi _i + S_1^{1,3}\delta _z \psi _i + S_0^1 \psi _i \, \mathrm {d}x\\ \underline{f}_S^{i,2}= & {} \int _{\varOmega } S_1^{2,1}\delta _x \psi _i + S_1^{2,2}\delta _y \psi _i + S_1^{2,3}\delta _z \psi _i + S_0^2 \psi _i \, \mathrm {d}x\\ \underline{f}_S^{i,3}= & {} \int _{\varOmega } S_1^{3,1}\delta _x \psi _i + S_1^{3,2}\delta _y \psi _i + S_1^{3,3}\delta _z \psi _i + S_0^3 \psi _i \, \mathrm {d}x, \end{aligned}$$
  where $S_0$ and $S_1$ are defined according to respectively (24) and (23).

The linear systems are assembled and manipulated using the PETSc (Balay et al. 2017) framework. All three linear system are solved using the Boomer (Henson and Yang 2002). Algebraic Multigrid preconditioner in combination with the GMRES solver provided by PETSc. The linear solver in the Newton method is configured with a relative residual error tolerance of $10^{-4}$, while all other solvers use an absolute residual error tolerance of $10^{-8}$. The detailed solver settings are listed in the appendix.

### Source localization

The goal of the source localization algorithm is to identify the midpoints $x_i$, $i=1,\ldots ,N$ of the sources $\{ \omega _i \}$ that minimize our functional *J*. Our shape calculus based on shape derivatives does not allow for splitting or the creation of activation sites. For this purpose one has to resort to topological derivatives.
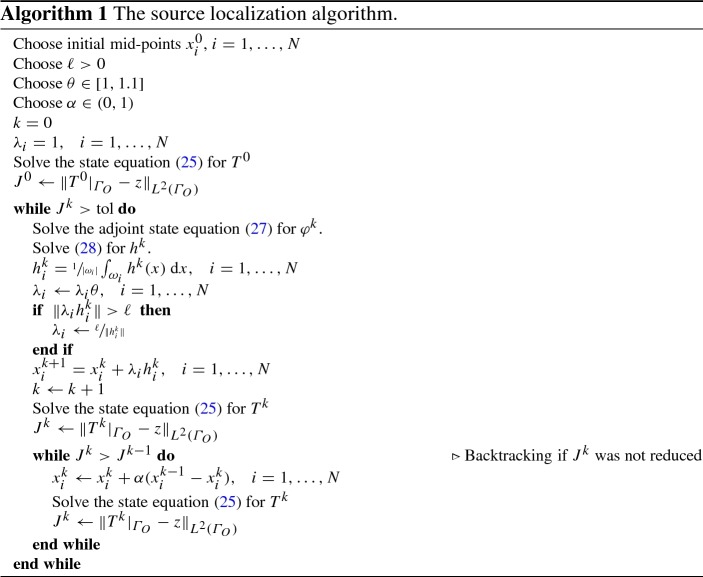


We propose the approach depicted in Algorithm 1. Required inputs are some starting locations $\{x_i^0\}$, a user-specified, mesh dependent step-length $\ell$ (usually 1-3 mesh edge-lengths), a step-length scaling parameter $\theta$ and a backtracking scale $\alpha$. The symbol $\Vert \cdot \Vert$ denotes the Euclidean norm. The algorithm starts by initializing $T^0$ and $J^0$. Then, while the tolerance condition on $J^k$ is not met, in each iteration of the while-loop it computes solutions to (27) and (28), updates the source midpoint positions and finally computes a new state solution to (25). If necessary backtracking is employed, and the next iteration begins.

For complex geometries, the step-length $\ell$ needs to be chosen small enough in order to prevent the sources from being moved out of $\varOmega$. Note, that $\ell$ only realizes an upper bound on $\Vert \lambda _i h_i^k \Vert$, but this quantity is not bounded from below. Choosing $\theta > 1$ improves convergence speed, as the $\lambda _i$ are scaled up to counteract the reduction of $h^k$. In the case of overshooting, oscillations are reduced by backtracking.

According to the problem statement, the sources $\{\omega _i\}$ are not part of the computational domain $\varOmega$. In each iteration *k*, all points of ${\bar{\varOmega }}$ are moved based on the perturbation field $h^k$, in particular the current source surface $\varGamma ^k = \cup _{i = 1}^N \omega _i^k$ is moved. In practice it is easier to solve also the state and adjoint equations on $U=\varOmega \cup {\bar{\omega }}$ with $\omega =\cup _{i=1}^N\omega _i^k$ and apply the Dirichlet boundary values on whole ${\bar{\omega }}$. Then we only translate the logical representation of $\omega$ and thus the discretization of *U* is not perturbed. This prevents the need for re-meshing and implicitly enables the merging of any $\omega _i$ without requiring special algorithmic treatment. Once $\Vert \lambda _i h_i^k\Vert$ is smaller than the average FE mesh edge-length, local refinement would become necessary. This however, is not within the scope of this work.

### Model parameters

The tensor parameter *M* contains the squared cardiac conduction velocity. In the depth of the human LV wall, conduction velocity is orthotropic due to numerous factors, with the most important ones being the geometry of myocytes and the non-uniform distribution of conduction-mediating proteins and sodium channels. The fastest propagation velocity $v_{\mathrm{f}}$ is observed along the prevailing long axis orientation of myocytes, often referred to as “fiber orientation” $\underline{f}$. Excitation spread within a sheet and along direction $\underline{s}$, which is orthogonal to $\underline{f}$, occurs at a lower conduction velocity $v_{\mathrm{s}}$, and even slower in a sheet normal direction $\underline{n}= \underline{f} \times \underline{s}$, at a velocity $v_{\mathrm{n}}$. Both orthotropic velocities as well as the principal axes $\{\underline{f}, \underline{s}, \underline{n}\}$ vary in space. In general, $v_{\mathrm{f}}> v_{\mathrm{s}} > v_{\mathrm{n}}$ holds where the ratios are assumed as $v_{\mathrm{f}}{:}\,v_{\mathrm{s}}{:}\,v_{\mathrm{n}} \approx 4{:}\,2{:}\,1$ based on experimental studies (Caldwell et al. 2009). As such, *M* is defined as

29 $$\begin{aligned} M := v_f^2 \, \underline{f} \otimes \underline{f} + v_s^2 \, \underline{s} \otimes \underline{s} + v_n^2 \, \underline{n} \otimes \underline{n}. \end{aligned}$$

The 2D benchmark in Sect. 4.2 will feature constant fiber-and sheet-directions $\underline{f}=(1,0)^*$ and $\underline{s}=(0,1)^*$ with varying $(v_f, v_s)$, while the 3D human LV benchmark in Sect. 4.3 will have constant velocities $v_f = 0.6 \, \hbox {m/s}$, $v_s = 0.4 \, \hbox {m/s}$, $v_n = 0.2 \, \hbox {m/s}$ and heterogeneous vectors $\{\underline{f}, \underline{s}, \underline{n}\}$, computed by a rule-based method (Bayer et al. 2012). Further, in the human LV benchmark *M*(*x*) is an element-wise function. This makes the computation of $S_0$ impractical. While it would be possible to change the representation of *M*, this has not been pursued, since the terms involving $S_0$ have only a small impact on the shape derivative, see the comparisons in Sect. 4.2.

The parameter $\varepsilon$ is calibrated by comparing the macroscopic velocity of propagating wavefronts generated by the viscous Eikonal model with physiological measurements such as the observed temporal delay between endocardial activation and epicardial breakthrough. Depending on a given trajectory relative to the used fiber field, macroscopic velocities fall into the range of local conduction velocities encoded in *M*, which themselves are based on experimental measurements (Caldwell et al. 2009).

## Evaluation benchmarks

Two numerical benchmarks, a 2D wedge benchmark and a 3D LV benchmark, will be used to evaluate the proposed algorithm’s ability to identify activation sources based on input boundary data.

### Evaluation criteria

In both benchmarks we measure both the convergence of the current source locations $\{x_i^k\}$ to the exact source locations $\{x_i\}$, and the reduction of the functional *J* defined in (1). Thus the following evaluation criteria are used:

- the distances to reference locations $d^k_i := \Vert x_i - x_i^k \Vert$
- the relative reduction $J^k / J^0$ with $J^k := \frac{1}{2}\int _{\varGamma _O}(T^k - z)^2 \mathrm {d}x$.

### 2D benchmark

In this benchmark, the computational domain *U* is given by the unit-square $(0,1)\times (0,1)$. We consider two activation sites $\omega _i=B_{0.1}(x_i)$ whose midpoints are given by $x_1=(0.5,0.3)^*$ and $x_2=(0.25,0.7)^*$. Thus we have $\varOmega =U{\setminus } \bigcup _{i=1}^2\omega _i$. The observed data are given on the boundary $\varGamma _N$ of *U*. The domain *U* is discretized by 66,049 vertices and 131,072 triangles, which yields a discretization size of $\approx 4\cdot 10^{-3}$. Moreover we set $g=0$, $f=1$, $\varepsilon =0.1$ and

$$\begin{aligned} M=\left( \begin{array}{cc} \sin (\pi x)+1.1 &{}\quad 0 \\ 0 &{}\quad \sin (\pi y)+1.1\\ \end{array} \right) . \end{aligned}$$

**Fig. 1:**
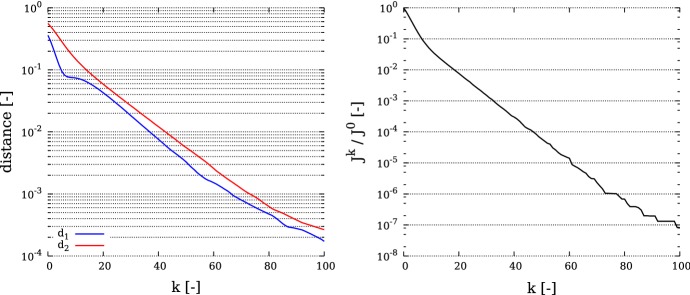
The evaluation criteria for the 2D benchmark. Left: distance to reference location $d^k_i := \Vert x_i^k-x_i\Vert , \,\, i=1,2$ over the iteration *k*. Right: relative functional reduction $J^k/J^0$ over the iteration *k*

**Fig. 2:**
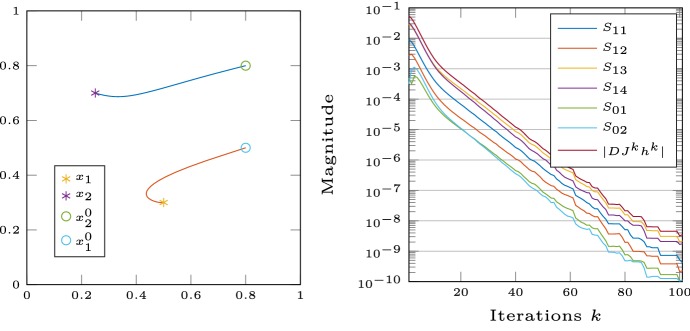
Left: trajectory of the points $x_1^k$ and $x_2^k$ during optimization. Right: magnitude of $S_{ij}$ over iterations *k*

**Fig. 3:**
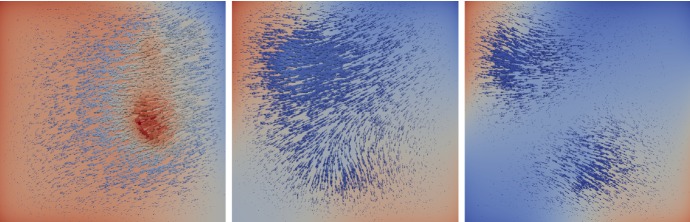
Perturbation field $h^k$ (arrows) and adjoint state variable $\varphi ^k$ (background color; blue-negative and red-positive) for $k=0,10,20$. The vectors are scaled for better visibility. The color of the vectors correlates with their length. (blue-short and red-long) (color figure online)

In this example we consider the noise free case. Thus the observed data *z* is generated by solving the state equation for *T* and restricting *T* to $\varGamma _N$. In Fig. 1 we observe that the distances between the exact midpoints $x_i$ and $x_i^k$ reach values below $10^{-3}$, more precisely $d_1=1.7\cdot 10^{-4}$ and $d_2=2.6\cdot 10^{-4}$, after 100 iterations. These distances correspond approximately to the mesh size. On the right of this figure we can note that $J^k/J^0$ attains a value of about $10^{-7}$. Figure 2 shows the trajectories of the points $x_i^k$ as the iteration proceeds. We can see that the midpoints $x_i^k$ do not move in straight lines. We expect that this is caused by interaction between the two activation sites, and the influence of *M*. Nevertheless the exact midpoints $x_i$ are reached with high precision. In Fig. 3 the perturbation field $h^k$ and the adjoint state $\varphi ^k$ are displayed for $k=0,10,20$. The dominant directions of the perturbation field point from regions of $\varOmega$ where $\varphi ^k$ is negative to regions of $\varOmega$ where $\varphi ^k$ attains high positive values. Moreover we see that the trajectories of the points $x_i^k$ (Fig. 2) correlate to the main directions of the perturbation field $h^k$.

In order to study the influence of the different parts of $DJ^k$ we introduce the quantities:

$$\begin{aligned} S_{11}= & {} \varepsilon \left| \int _\varOmega (\mathrm {Id}_{{\mathbb {R}}^d}M\nabla T^k\cdot \nabla \varphi ^k){:}\,Dh^k~\mathrm {d}x\right| , \\ S_{12}= & {} \left| \int _\varOmega (|\nabla T^k|_M^2-1)\varphi ^k\mathrm {Id}_{{\mathbb {R}}^d}){:}\,Dh^k~\mathrm {d}x\right| , \\ S_{13}= & {} \varepsilon \left| \int _\varOmega (\nabla T^k\otimes M\nabla \varphi ^k+\nabla \varphi ^k\otimes M\nabla T^k){:}\,Dh^k~\mathrm {d}x\right| , \\ S_{14}= & {} \left| \int _\varOmega (2\nabla T^k\otimes M\nabla T^k\varphi ^k){:}\,Dh^k~\mathrm {d}x\right| ,\\ S_{01}= & {} \varepsilon \left| \int _\varOmega (M_{\nabla T^k}^*\nabla \varphi ^k)\cdot h^k~\mathrm {d}x\right| \quad \text {and}\\ S_{02}= & {} \left| \int _\varOmega (M_{\nabla T^k}^*\nabla T^k\varphi ^k)\cdot h^k~\mathrm {d}x\right| . \end{aligned}$$

We clearly see in Fig. 2 that $S_{13}$ and $S_{14}$ are the dominating summands in $|DJ^kh^k|$. Thus it is justified to omit the terms $S_{01}$ and $S_{02}$ in the following benchmark. We also carried out tests with different choices for the conductivity tensor *M* and found nearly identical behavior provided that *M* is given by (29) with orthonormal vectors $\underline{f}$ and $\underline{s}$. If the choice for *M* violates the orthonormality condition for $\underline{f}$ and $\underline{s}$, then the numerical results may depend on the directions determined by the spatial relation between the exact activation sites and the initial guess, and the directions given by $\underline{f}$ and $\underline{s}$.

### 3D LV benchmark

The 3D LV benchmark serves to gauge the potential of the proposed method in an envisioned clinical application which is geared towards localizing earliest activation sites from epicardial activation maps. In line with early experimental mapping studies (Durrer et al. 1970) on ex vivo human hearts we assume that there are three discrete sites of earliest activation located at the endocardial surface of the LV. In anatomical terms, these sites are located higher towards the base of the LV on the anterior paraseptal wall, a central area at the septal endocardium, and a posterior paraseptal area. Therefore the conduction system activating the LV is referred to as “*trifascicular*” with the three fascicles being referred to as anterior fascicle $x_{\mathrm {af}}$, posterior fascicle $x_{\mathrm {pf}}$ and septal fascicle $x_{\mathrm {sf}}$. Each of these fascicles can be considered as a patch of tissue composed of a tight network of Purkinje fibers which are electrically coupled to the LV myocardium through so-called Purkinje-Ventricular junctions (PVJs). Owing to the fast conduction properties of the Purkinje fibers in these patches a large number of PVJs are located which activate one after the other with very short delays. Thus, these patches appear to activate simultaneously and are considered a fascicle and not a large set of individual PVJs. Further, due the short delays and the close spatial vicinity, it is still highly challenging today, even with invasive mapping devices recording signals with electrodes located in the immediate vicinity of PVJs, to identify individual PVJs. As such we do not expect that the identification of individual PVJs using data recorded at the epicardial surface is feasible.

While the presence of three fascicles is widely accepted and there general location is assumed to be known, the inter-individual variability and their exact location, size and relative timing is significant. Based on these considerations we assume the activation map (either measured or precomputed) on the epicardial surface as given input data for the localization of the three LV fascicles which we deem a plausible and sufficiently accurate general representation of the actual activation sources. Moreover, we simplify by assuming size and timing of individual fascicles as given and focus only on the identification of their location.

**Fig. 4:**
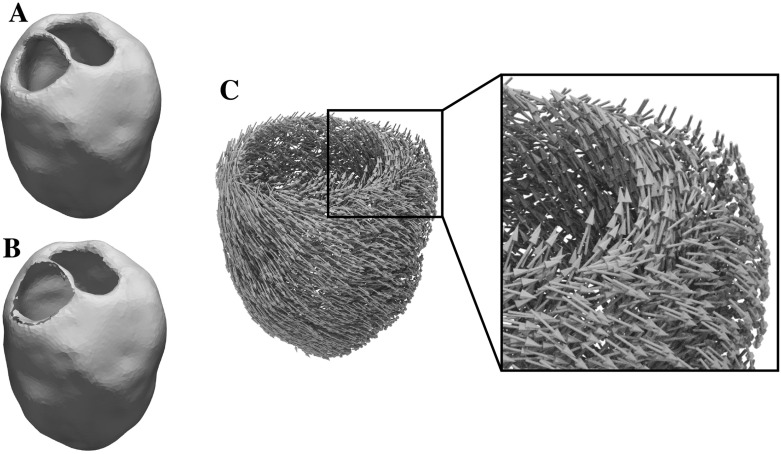
**a** The LV geometry forming $\varOmega$, **b** the surface $\varGamma _O$, **c** the fiber directions $\underline{f}$

The discretized model of a human LV forming the computational domain $\varOmega$ consists of 47,938 vertices and 245,611 tetrahedra, with an average discretization size of $\approx 1.5\,\hbox {mm}$. The observable surface $\varGamma _O$ is formed by the epicardial surface of the LV. We refer to Fig. 4. The source surface is $\varGamma = \cup _{i=1}^3 \partial \omega _i$ with $\omega _i := B_{r=3\hbox {mm}}(x_i), \, i=1,2,3$. Further, based on numerical tests with varying activation sequences we chose $\varepsilon = 80\,\hbox {ms}$ to obtain appropriate macroscopic conduction velocities which fall into the range of local conduction velocities encoded in *M* (see Sect. 3.5).

**Fig. 5:**
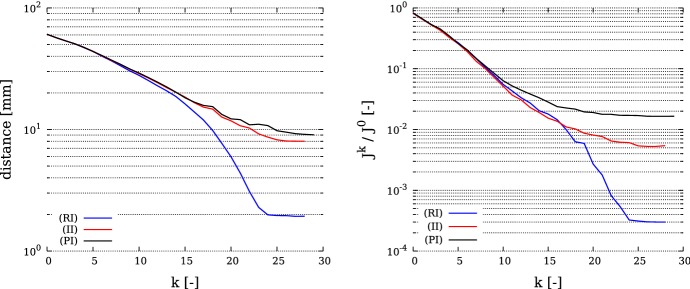
The evaluation criteria for the cases (RI), (II) and (PI) of the LV benchmark. Left: $\sum _{i=1}^3 d^k_i$ over the iteration *k*. Right: Relative functional reduction $J^k / J^0$ over the iteration *k*

Motivated by real-world applications, we want $z \in L^2(\varGamma _O)$ to correspond to some error-prone data defined on a lower spatial resolution than the computational resolution of $\varGamma _O$. To accommodate for this, the data *z* are generated as follows:

1. A reference activation time $T^r$, using the source locations
  $$\begin{aligned} x_1= & {} (60.3,27.6,-20.9)^*,\quad x_2=(42.7,-12.8,-2.6)^*\\&\quad \text {and}~x_3=(26.9,19.6,-39.1)^* \end{aligned}$$
  is computed.
2. $T^r$ is sub-sampled at a set of 106 uniformly spaced sample points $\{s_i\}_{i=1}^{106} \in \varGamma _O$ yielding $t_i := T^r(s_i)$.
3. A zero-average uniform noise is added: $t_i \leftarrow t_i + \xi _i \, \, 1/|\varOmega |\int _{\varOmega } T^r(x) \mathrm {d}x, \,\, i=1,\ldots ,106$ with $\xi _i \in [-\xi /2, \xi /2]$.
4. The data *z* is interpolated from the perturbed samples $\{t_i\}$ using distance-weighted interpolation.

We compare the following cases for different data selections:

- **(RI)** Using the reference data as input: $z := T^r|_{\varGamma _O}$.
- **(II)** Using only interpolated input: *z* is generated as described above with $\xi = 0$.
- **(PI)** Using perturbed and interpolated input: *z* is generated as described above with $\xi = 0.3$.

Starting at the initial locations

$$\begin{aligned} x_1^0= & {} (71.1,11.3,-18.4)^*,\quad x_2^0=(44.7,-23,-25.2)^*\\&\quad \text {and}~x_3^0=(42.3,9.1,-46.8)^*, \end{aligned}$$

the source localization Algorithm 1 is applied. In order to find the best achievable results, the algorithm is configured to only stop if *J* cannot be further reduced. All three cases executed in approximately 250 seconds on 10 cores of a workstation PC with two Intel Xeon E5645 (2.40 GHz) CPUs.

Figure 5 shows the two evaluation criteria—the summed distances to reference location and the relative reduction of *J*—over the iteration count. For (RI), the algorithm terminates after 29 iterations with a relative error minimum of $3 \cdot 10^{-4}$. The highest final distance to reference location is $d_1 = 1\,\hbox {mm}$, which is well below the average FE edge-length of $1.5\,\hbox {mm}$. The discrete representation of the reconstructed activation sites closely match the desired reference sites.

**Fig. 6:**
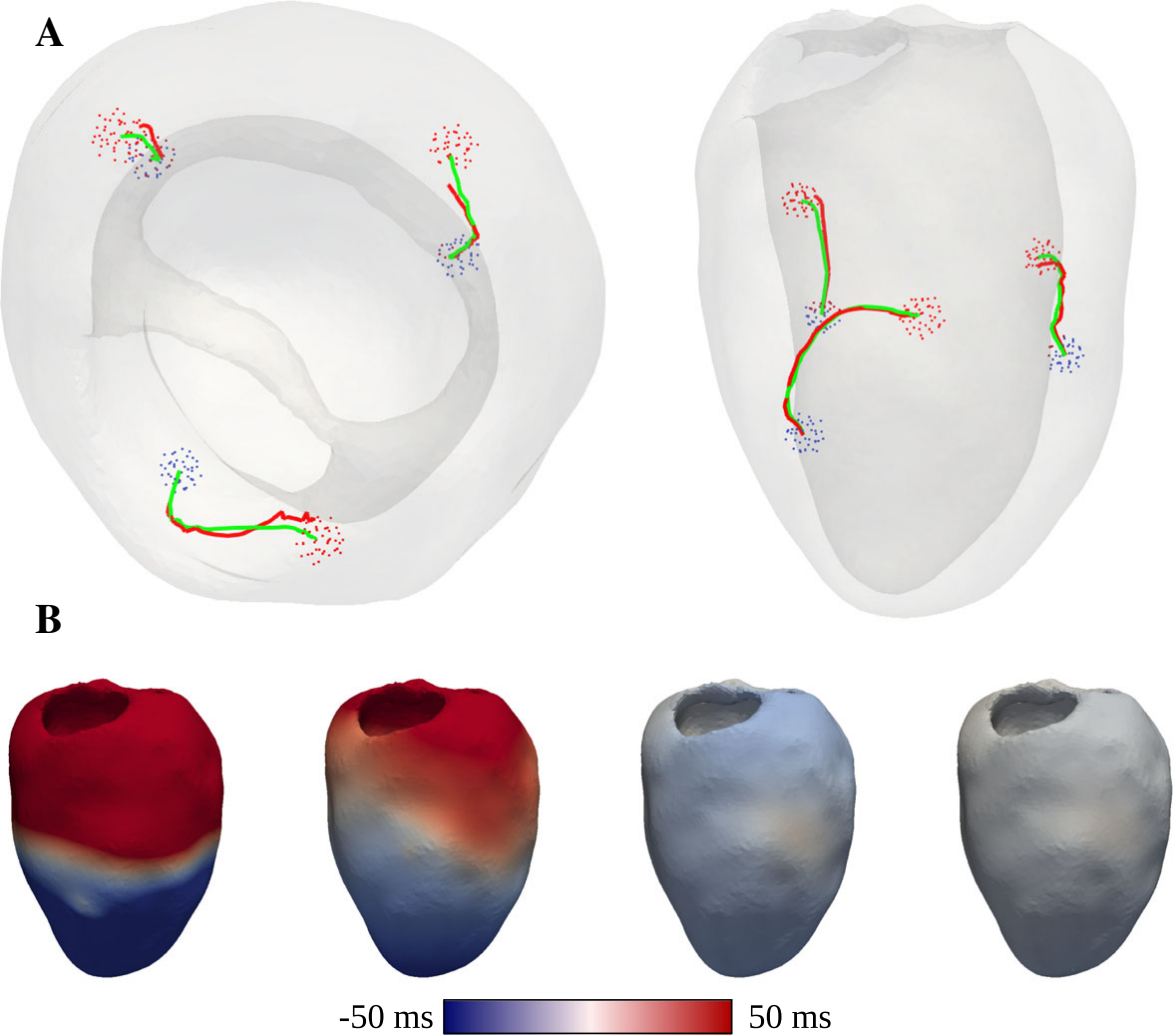
**a** The trajectories traveled by $x_i^k$ for the (RI) and (PI) cases. The (RI) trajectory is colored in green, while the one of the (PI) case is colored in red. The mesh vertices inside ball $\omega _i$, used for the reference solution $T^r$, are displayed in red, while those inside the initial search ball $\omega _i^0$ are displayed in blue. **b** The adjoint solution $\varphi ^k$ for the (RI) case, for the iterations $k = 0, 10, 20, 28$, respectively from left to right (color figure online)

In the (II) case, the algorithm stops after 29 iterations. The minimal relative error is $5.4 \cdot 10^{-3}$. The highest final distance is $d_1 = 4.3\,\hbox {mm}$, which is significantly larger than in the (RI) case. This indicates that the low-resolution sampling of $T^r|_{\varGamma _O}$ lowers the quality of our reconstruction. Also, the interpolation induces noise which impairs the reconstruction quality.

For (PI), the algorithm terminates after 30 iterations with relative error $1.6 \cdot 10^{-2}$. The $d_i$ are similar to the (II) case, although slightly higher, with the highest final distance $d_1 = 4.4\,\hbox {mm}$. This further hints that the low-resolution sampling has a much greater effect on the source locations than the error due to noise.

For all three cases, the final displacements $x_i^k - x_i^{k-1}$ are smaller than 0.2 mm, and therefore only a fraction of the mesh edge-length of 1.5 mm. As such, some mesh manipulation (e.g. mesh refinement, mesh deformation) would be necessary in order to apply the source displacement on the state and adjoint state problems. Since the mesh is not adjusted in the presented paper, this leads to a stagnation of the algorithm.

**Fig. 7:**
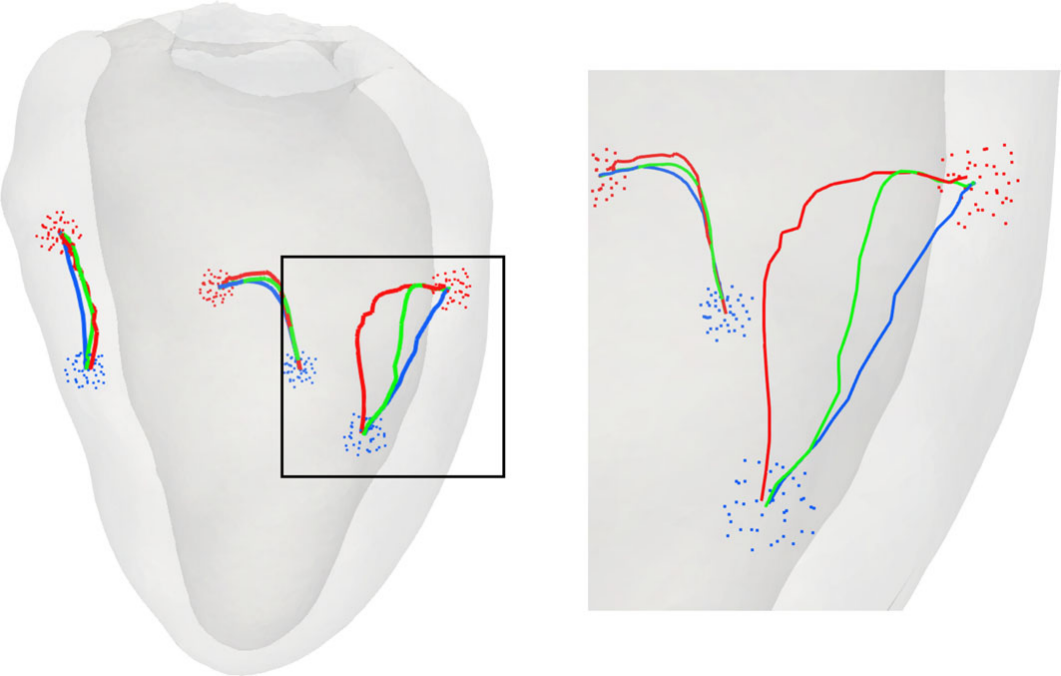
The source trajectories in the (RI) case for three different choices of *M*. Green: $v_f = 0.6, v_s = 0.4, v_n = 0.2$. Blue: $v_f = 0.5, v_s = 0.5, v_n = 0.5$. Red: $v_f = 0.8, v_s = 0.4, v_n = 0.2$ The mesh vertices inside ball $\omega _i$, used for the reference solution $T^r$, are displayed in red, while those inside the initial search ball $\omega _i^0$ are displayed in blue (color figure online)

Figure 6 visualizes the source localization process by displaying the trajectories of $x_i^k$ and the adjoint solution $\varphi ^k$ during the source localization process. By comparing figure parts A and B, we observe that the motion induced by the field *h* is oriented from negative to positive regions of $\varphi ^k$, similar to the 2D benchmark in Sect. 4.2. Further, we see the diminishing absolute values of $\varphi ^k$ over the iteration count. The final locations in Fig. 6a show, that even the worst localization (PI) still offers a good approximation of the general source location, well inside the uncertainty bounds of clinical parameters. Moreover, we carried out numerical tests with varying anisotropy ratios, see Fig. 7. In the LV benchmark, the convergence trajectory of one source varied significantly between the three choices of *M*. Numerical tests with significantly higher anisotropy ratios indicate, that a higher FE mesh resolution is required, particularly in the case of large displacements orthogonal to $\underline{f}$.

## Discussion

This study presented analysis and implementation of an algorithm for identifying sites of earliest activation in the LV from epicardial activation maps. The algorithm is posed as an optimization problem, where initial activation sites are chosen first to be then iteratively perturbed in order to minimize the mismatch between computed activation times and the activation maps given at the epicardial surface. We demonstrated well-posedness of all sub-problems, namely the viscous Eikonal equation, the tangent and adjoint equations and the perturbed state equation and characterized the shape derivative.

The theoretical results were verified by solving two benchmark problems, a 2D unit-square benchmark and a 3D human LV benchmark. For unperturbed input data, the localization method was able to accurately reconstruct the sites of initial activation. The largest deviations observed were $2.6\cdot 10^{-4}$ and 1 mm, respectively, for the 2D and 3D benchmark. This was significantly smaller than the respective spatial discretization sizes of $4\cdot 10^{-3}$ and 1.5 mm used in 2D and 3D benchmark, respectively. To probe the robustness of the method, the 3D benchmark was repeated using input data of reduced quality, that is, epicardial activation were spatially under-sampled and noise was added. These benchmark results showed, that the identification of earliest activation sites was still feasible, yielding a sufficiently accurate approximation of the general locations, comparable or better than the accuracy achieved with clinically used invasive endocardial mapping systems (Gepstein et al. 1997).

Several topics suggest themselves as possible extensions of the present work. The shape gradient is already set up to allow for a more realistic representation of the activations sites than those considered in the numerical realizations of these first benchmarks. Also, it can be of interest to incorporate different activation times by introducing inhomogeneous Dirichlet boundary conditions with unknown forcing terms. To allow for additional accuracy of the reconstruction of the evolution of the activation regions local grid refinement can be considered in future algorithmic efforts. Further it can be an interesting task to carry out the asymptotic analysis for $\varepsilon \rightarrow 0$.

### Limitations

While the benchmarks in this study demonstrate that the identification of sites of earliest endocardial activation from epicardial activation maps is, in principle, feasible with the proposed method, with regard to practical applications a number of restrictions apply. Out method makes various tacit assumptions which may not always hold in practice. Fiber arrangements are assumed to be known, following largely the patterns observed experimentally in the healthy LV (Streeter et al. 1969). With current technology fiber arrangements cannot be measured in vivo with sufficient spatial resolution,but suitable technologies under development (Scott et al. 2018) promise to lift this restriction in the future. Further, conduction velocities along the principal tensor axes were also assumed homogeneously throughout the LV, as velocities cannot be determined accurately in vivo, the chosen values were based on experimental observations (Caldwell et al. 2009). These values and their ratios may deviate from the experimentally estimation of $v_{\mathrm{f}}{:}\,v_{\mathrm{s}}{:}\,v_{\mathrm{n}}=3{:}\,2{:}\,1$, and they may not be constant throughout the myocardium. Identifying the velocity tensor fields is therefore an additional complexity which is a related research topic (Marchesseau et al. 2013a) that has not been addressed in this study. A further limitation is the assumption that three sites of earliest endocardial activation exist. While this is physiologically motivated based on the notion that three main fascicles initiate activation in the healthy human LV endocardium (Durrer et al. 1970), this may not always be the case, particularly not under pathological conditions such as a left bundle branch block where the electrical activation of the LV may follow a markedly different pattern.

## Appendix

### PETSc solver options

The following solver configuration parameters were passed at run-time to PETSc:

### Units

The results of the LV benchmark use the following units:

**Table:**

Variable	Unit
$T, \varphi$	ms
*h*	mm
*M*	$\hbox {mm}^{2}/\hbox {ms}^2$
$\varepsilon$	ms
